# Full-fat dairy products and cardiometabolic health outcomes: Does the dairy-fat matrix matter?

**DOI:** 10.3389/fnut.2024.1386257

**Published:** 2024-07-29

**Authors:** Victoria M. Taormina, Allison L. Unger, Jana Kraft

**Affiliations:** ^1^Department of Animal and Veterinary Sciences, The University of Vermont, Burlington, VT, United States; ^2^Department of Pathology and Laboratory Medicine, The University of Vermont, Burlington, VT, United States; ^3^National Dairy Council, Rosemont, IL, United States; ^4^Department of Medicine, Division of Endocrinology, Metabolism, and Diabetes, The University of Vermont, Colchester, VT, United States; ^5^Department of Nutrition and Food Sciences, The University of Vermont, Burlington, VT, United States

**Keywords:** fatty acids, milk fat globule membrane, obesity, type 2 diabetes, cardiovascular diseases, hyperglycemia, insulin resistance, inflammation

## Abstract

Reducing dairy fat intake is a common dietary guideline to limit energy and saturated fatty acid intake for the promotion of cardiometabolic health. However, research utilizing a holistic, food-based approach to assess the consumption of the fat found in dairy, a broad and diverse food group, may provide new insight into these guidelines. Dairy fat is comprised of a diverse assembly of fatty acids, triacylglycerols, sterols, and phospholipids, all uniquely packaged in a milk fat globule. The physical structure of this milk fat globule and its membrane is modified through different processing methods, resulting in distinctive dairy-fat matrices across each dairy product. The objectives of this narrative review were to first define and compare the dairy-fat matrix in terms of its unique composition, physical structure, and fat content across common dairy products (cow’s milk, yogurt, cheese, and butter). With this information, we examined observational studies and randomized controlled trials published within the last 10 years (2013–2023) to assess the individual effects of the dairy-fat matrix in milk, yogurt, cheese, and butter on cardiometabolic health and evaluate the implications for nutrition guidance. Searches conducted on Ovid MEDLINE and PubMed^®^ utilizing search terms for cardiometabolic health, both broadly and regarding specific disease outcomes and risk factors, yielded 59 studies that were analyzed and included in this review. Importantly, this review stratifies by both dairy product and fat content. Though the results were heterogeneous, most studies reported no association between intake of these individual regular-fat dairy products and cardiometabolic outcome measures, thus, the current body of evidence suggests that regular-fat dairy product consumption may be incorporated within overall healthy eating patterns. Research suggests that there may be a beneficial effect of regular-fat milk and yogurt intake on outcome measures related to body weight and composition, and an effect of regular-fat cheese intake on outcome measures related to blood lipids, but more research is necessary to define the directionality of this relationship. Lastly, we identify methodological research gaps and propose future research directions to bolster the current evidence base available for ascertaining the role of dairy fat in a healthy diet.

## 1 Introduction

Dairy products are a prominent dietary constituent globally, as evidenced by their inclusion in food-based dietary guidelines across the world (1). Yet, these guidelines predominantly advise reducing dairy fat intake, via the consumption of primarily low-fat or fat-free dairy products, to align with other common recommendations to reduce energy and specifically the intake of saturated fatty acids (SFAs), given the high SFA content in dairy fat (1–3). However, this reductionist approach—the concept that one single nutrient or nutrient class (e.g., SFAs) leads to a certain health effect (e.g., cardiovascular diseases; CVD)—is being challenged with an emerging body of research demonstrating the influence of the food matrix, i.e., the interplay of a food’s physical structure and nutritional and bioactive composition (4–7). Dairy products exemplify the concept and importance of the food matrix as the milk, yogurt, cheese, and butter available on the market are all derived from raw milk, but have key differences such as physical structure, nutrient content and composition, as well as potentially different physiologic effects (4, 8, 9), raising the question as to whether the current body of science on cardiometabolic health effects of dairy fat is reflected in current nutrition guidance.

This narrative review considered the differences between commonly consumed dairy products (cow’s milk, yogurt, cheese, and butter) in terms of fat content, composition, and physical structure with the goal to examine and distinguish their roles in cardiometabolic health. Our aims were to: (i) define the dairy-fat matrix, (ii) describe the unique fat matrices of milk, yogurt, cheese, and butter, and (iii) examine the effect of the dairy-fat matrix of these dairy products on cardiometabolic health outcomes by stratifying by both dairy product and fat content.

## 2 Dairy fat physical structure and composition

Dairy fat is the most complex edible fat in nature and comprises three different levels of lipid organization with increasing complexity. The following section outlines this complexity, starting with the fundamental unit of lipids, the fatty acids (FAs), then progressing to the combination of FAs with other moieties (e.g., glycerol) to produce the macromolecules triacyglycerols (TAGs), phospholipids, and sterols, then finally the formation of the milk fat globule (MFG), a vesicle specific to milk and milk-derived dairy products, using these macromolecules. Understanding the complexity of the dairy-fat matrix is central to understanding the differences between dairy products and may be a key mechanistic consideration for their effects on cardiometabolic health.

### 2.1 Fatty acids

**Figure 1:**
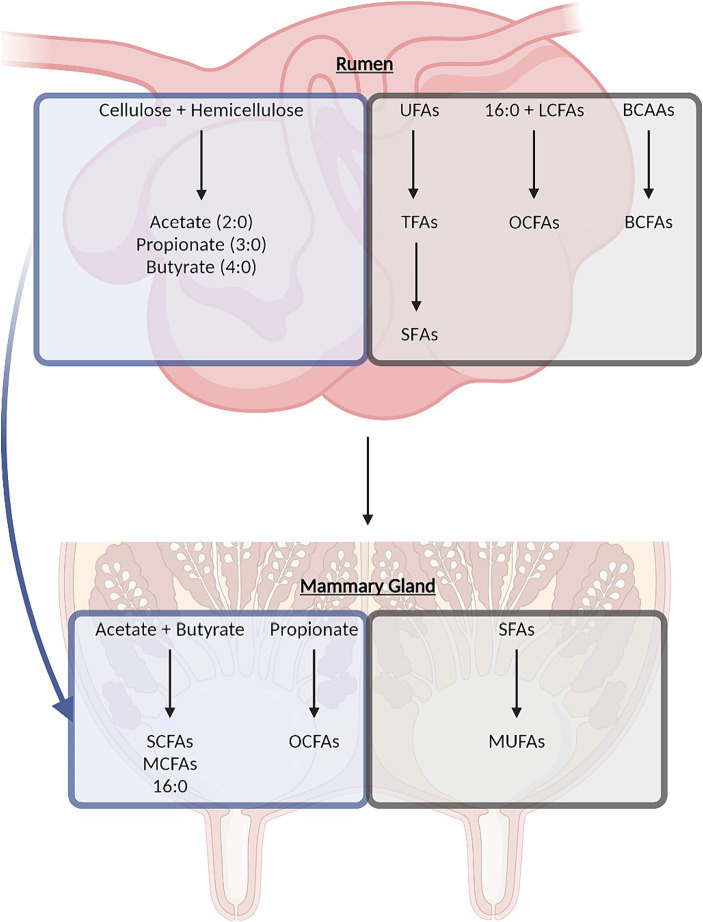
Overview of dairy-derived fatty acid synthesis in the rumen and mammary gland through de novo synthesis (blue boxes) or synthesis from dietary components (gray boxes). BCAAs, branched-chain amino acids; BCFAs, branched-chain fatty acids; LCFAs, long-chain fatty acids; MCFAs, medium-chain fatty acids; MUFA, monounsaturated fatty acids; OCFAs, odd-chain fatty acids; SCFAs, short-chain fatty acids; SFA, saturated fatty acids; TFAs, trans-fatty acids; UFAs, unsaturated fatty acids.

At least 400 different FAs have been identified in dairy fat including even-chain FAs and odd-chain FAs (OCFAs), cis-FAs and trans-FAs, short-chain FAs (SCFAs), medium-chain FAs (MCFAs), and long-chain FAs (LCFAs), straight-chain FAs and branched-chain FAs (BCFAs), as well as SFAs, monounsaturated FAs (MUFAs), and polyunsaturated FAs (PUFAs) (see Supplementary Table S1 for proportions of select abundant FAs). SFAs constitute approximately 68% of total FAs as the predominant FA class in dairy fat, followed by MUFAs at approximately 27% and relatively small amounts of PUFAs at approximately 4% (10); these percentages can vary widely based on a variety of factors including, but not limited to, diet, genetics, or stage of lactation (11). Palmitic acid (16:0), oleic acid (18:1 c9), and linoleic acid (18:2 c9, c12) are the individual FAs that make up the major proportion of the SFA, MUFA, and PUFA classes, respectively. The complexity of the dairy FA composition is the result of the extensive lipid metabolism occurring throughout the dairy cow (12, 13). Dairy FAs originate from both de novo synthesis and preformed FAs (i.e., FAs from feed, endogenous and microbial metabolic pathways, and microbial membranes; Figure 1). Starting with de novo synthesized FAs, ruminal bacteria ferment cellulose and hemicellulose to acetate (2:0), propionate (3:0), and butyrate (4:0), which can be taken up by various tissues for additional metabolic processing (12, 14–16). Acetate and butyrate give rise to even-chain SFAs with four to 14 carbons and half of palmitic acid in milk through de novo synthesis in the mammary gland (13, 14). In adipose tissue, propionate can be used to synthesize OCFAs, which can, in turn, be transported to the mammary gland for incorporation into milk fat, although this pathway may also occur directly in the mammary gland (16). Preformed FAs are derived from components of the cow’s diet; some appear in the milk in their original form, whereas others are transformed by endogenous or microbial metabolism. Even-chain SFAs with 18 or more carbons and half of the palmitic acid in milk are directly derived from the lipids in the diet or mobilized from adipose tissue during certain energy states (11, 13, 14, 17–19). Several MCFAs and LCFAs can be desaturated in the mammary gland or in the adipose tissue or liver prior to transport to the mammary gland (11, 13, 14). Dietary unsaturated FAs, namely oleic acid, linoleic acid, and α-linolenic acid (18:3 c9, c12, c15), are readily biohydrogenated by bacteria in the rumen, producing various trans-18:3, trans-18:2, trans-18:1 isomer intermediates, including vaccenic acid (18:1 t11), and rumenic acid (18:2 c9, t11), as well as stearic acid (18,0) as the major biohydrogenation end product (13, 19, 20). Rumen biohydrogenation has been described in greater depth by Shingfield and Wallace (19). Bacterial and protozoal species in the rumen also convert dietary branched-chain amino acids (valine, leucine, and isoleucine) or endogenously produced keto-acids (isovaleric acid, 2-methylbutyric acid, isocaproic acid, and isobutyric acid) to BCFAs for incorporation into their membranes (21). Rumen bacteria and protozoa may also produce membrane OCFAs through α-oxidation of LCFAs (16, 22). During digestion, a portion of rumen microbial cells passes out of the rumen to the small intestine where membrane FAs are taken up and transported for incorporation into the milk (23–25).

### 2.2 Triacyglycerols, phospholipids, and sterols

**Figure 2:**
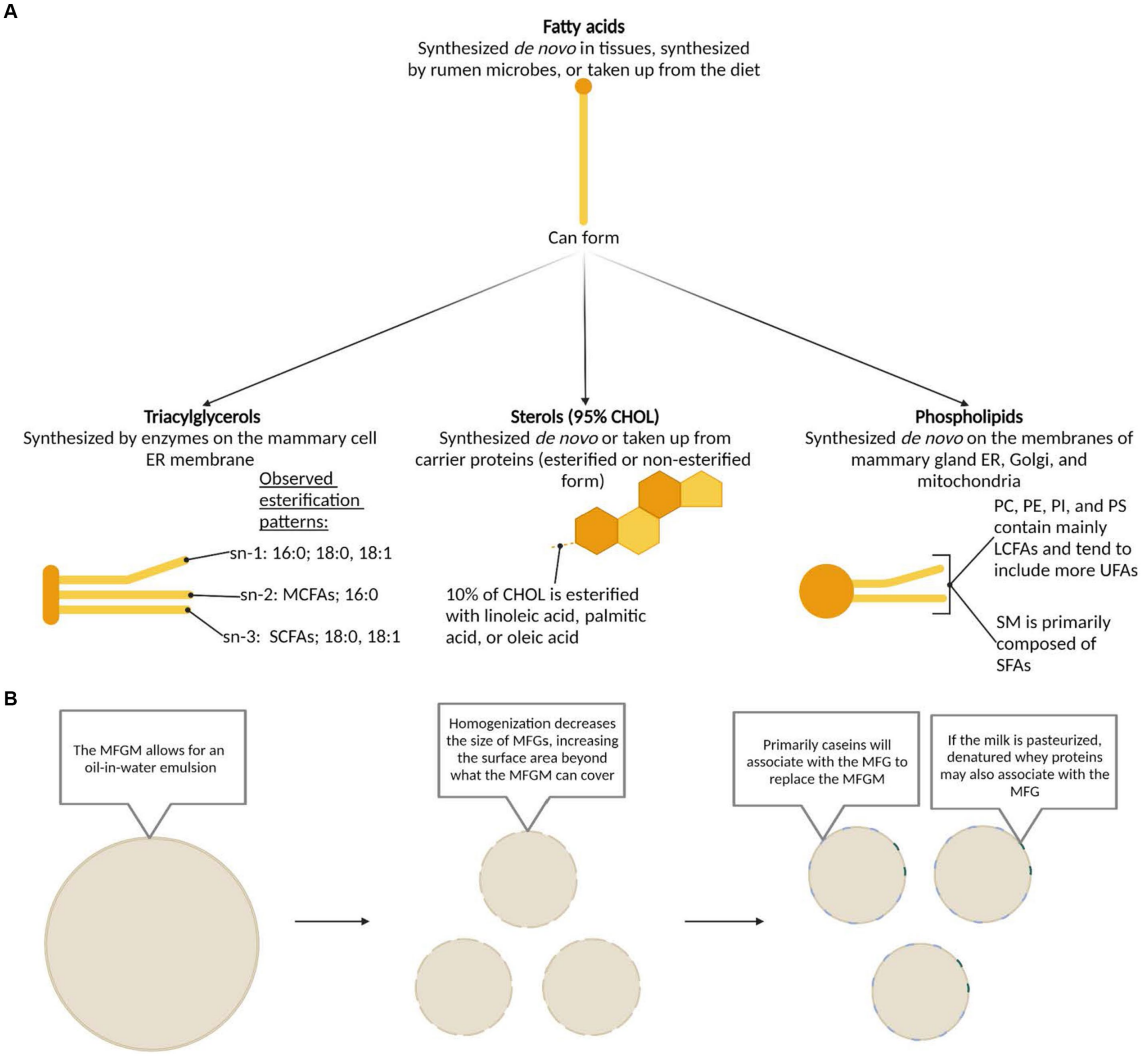
**(A)** Synthesis pathways, structures, and esterification patterns of triacylglycerols, sterols, and phospholipids formed from dairy fatty acids. Based on previously published work: fatty acids (12, 27); triacyclglycerols (12, 27, 28); sterols (12, 29, 30); phospholipids (13, 31, 32). **(B)** Structure of the milk fat globule membrane in milk before and after common processing methods. CHOL, cholesterol; ER, endoplasmic reticulum; LCFA, long-chain fatty acid; MCFA, medium-chain fatty acid; MFG, milk fat globule; MFGM, milk fat globule membrane; PC, phosphatidylcholine; PE, phosphatidylethanolamine; PI, phosphatidylinositol; PS, phosphatidylserine; SCFA, short-chain fatty acid; SM, sphingomyelin; UFA, unsaturated fatty acid.

Beyond the complex array of FAs in dairy fat, this complexity multiplies as they are combined to form the secondary structures of TAGs, phospholipids, and sterols. In milk, 97–98% of FAs are found in the form of TAGs, with approximately 1% as phospholipids, and less than 1% each as sterols and free FAs (14). TAGs are categorized by their number of carbons, with 26–56 carbon TAGs as the most prevalent, while phospholipids are categorized by their polar head, with phosphatidylcholine (PC), phosphatidylethanolamine (PE), and sphingomyelin (SM) as the most prevalent (26). Cholesterol is the primary sterol, comprising over 95% of the sterol class (26). Each of these lipid classes can be synthesized *de novo* in the mammary gland, though sterols are also produced in other tissues and are taken up by the mammary gland from plasma lipoproteins (Figure 2A) (12, 13, 27–32).

### 2.3 Native milk fat globule

Dairy fat is physically arranged in the form of a MFG surrounded with a distinct MFG membrane (MFGM), making it unique from other commonly consumed lipid sources. These MFGs range from 0.1 to 20 μm in diameter and the MFGM itself ranges from 8 to 10 nm in width (33, 34). An inner, central, and outer layer constitute the MFGM. The inner layer is comprised of polar lipids, derived from the endoplasmic reticulum of the mammary cell, that surrounds the central core of TAGs (35). The central layer is particularly dense, as it predominantly comprises of proteins from the cytoplasm (36, 37). The outer polar lipid bilayer is a concentrated layer of phospholipids predominantly comprised of glycerophospholipids and sphingolipids, which are derived from the cell membrane of the mammary cell (36). PE, phosphatidylinositol (PI), and phosphatidylserine (PS) tend to be located on the inner surface while PC and SM largely occur on the outer bilayer (38, 39). Transmembrane proteins, cholesterol (CHOL), glycoproteins, and enzymes, along with lipid rafts comprised of concentrated sphingomyelin and CHOL also appear on the outer layer, highlighting the influence of the unique amphipathic properties of these polar lipids (33, 34, 40, 41). The MFGM prevents the aggregation of MFGs, creating an emulsion within the milk (12) and protecting the inner TAG core from enzymatic degradation and oxidation (27). For detailed visualizations of the MFG and MFGM, we recommend the following publications (42–44).

## 3 Characteristics of fat in processed milk, yogurt, cheese, and butter

With an understanding of the FA composition and lipid classes present in unprocessed (i.e., raw) milk, we investigated the similarities and differences between the dairy products milk, yogurt, cheese, and butter based on their fat content, composition, and physical structure.

### 3.1 Dairy fat composition and content

The FA composition of milk is well documented, with comparatively less data available for yogurt, cheese, and butter (Supplementary Table S1). Overall, the percentages of FAs are similar among these dairy products while the fat content varies greatly. As listed on FoodData Central, a U.S. Department of Agriculture (i.e., USDA) food composition database of comprehensive food nutrient profiles, regular-fat (whole) milk and yogurt contain relatively low amounts of total fat [3.2 g per 100 g (45, 46)], compared to regular-fat cheese and butter [15.5–34 g per 100 g (47–68) and 82.2 g per 100 g (69), respectively], with butter having the highest total fat content. The large range of total fat content in regular-fat cheeses is due to the vast variety of cheeses available, each with key differences resulting from the unique milk base (i.e., pasteurized and/or homogenized milk), heating, acidification, salting, pressing, and/or ripening methods used (70). Among the cheeses surveyed on FoodData Central, paneer [15.5 g fat per 100 g (60)], mozzarella [20.4 g fat per 100 g (68)], and queso fresco [23.8 g fat per 100 g (58)] contained the lowest fat contents and Colby [32.1 g fat per 100 g (47)], gruyere [32.3 g fat per 100 g (53)], and cheddar [34 g fat per 100 g (62)] contained the highest fat contents, comparatively.

### 3.2 Dairy-fat matrix

The processing methods utilized in the dairy industry create different dairy-fat matrices between milk, yogurt, cheese, and butter. Within the food matrices of dairy products that encompass all the critical components in dairy is the dairy-fat matrix, which refers to the unique packaging and compartmentalization of the variety of lipids, as well as carbohydrates and proteins, found within and surrounding the MFG, and the specific physical and chemical interactions between these and other nutrients present. The physical structure of the MFG in these dairy products is modified by processing methods, resulting in different dairy-fat matrices and, likely, different physiologic and metabolic characteristics. Below, we provide an overview of the dairy-fat matrix in milk, yogurt, cheese, and butter for context, however, the specific metabolic implications of the alterations to the dairy-fat matrix are reviewed elsewhere (30, 71, 72).

#### 3.2.1 Dairy-fat matrix alterations from milk processing

Unprocessed milk is comprised of an oil-in-water emulsion where the MFGM and its protected fat droplets are suspended in the aqueous phase of milk (Figure 2B) (28, 72, 73). During homogenization, the MFG size is substantially reduced, leading to an overall increase in surface area of the MFGs (74–77). Subsequently, casein primarily aggregates and covers the new surface area (30, 36, 72, 78), and thereby increases the width of the MFG (73, 79). During pasteurization, denatured whey proteins, primarily β-lactoglobulin, aggregate with the proteins of the MFGM and the caseins on the MFG (30, 74). In the case pasteurization is performed prior to homogenization, there may be more whey proteins on the surface of the MFG, along with whey-casein complexes (30). Most authors report no change in MFG size in response to pasteurization (77, 80–82), though Ren et al. (83) described an increase in MFG size as a result of heat treatment.

#### 3.2.2 Dairy-fat matrix alterations from yogurt and cheese processing

During the production of yogurt or cheese, the coagulation of milk creates a semi-solid milk gel with MFGs interspersed in a casein protein network (76, 84, 85). The size of the MFGs and their associations with the protein network greatly impact the final physical structure, and therefore texture, of these products. Homogenized milk is frequently used as a base product for yogurt and softer cheeses as the smaller MFGs allow for better moisture retention, whereas unhomogenized milk is more commonly used for hard and semi-hard cheeses (86–88). MFGs that are larger than the pores of the protein network disrupt the network structure, while MFGs that are smaller than the pores act as inert fillers of this network (89). However, smaller MFGs may support the network structure if there are caseins present on the MFGM, such as when homogenized milk is the base product, as the caseins can interact with other proteins in the network (70, 84). In addition, it is common to observe the coalescing or aggregation of MFGs as a result of mechanical, thermal, or enzymatic processing techniques (11, 84). Notably, the high calcium content of cheese affects the dairy-fat matrix, as the interplay between milk calcium and caseins impacts the formation of the protein network in which MFGs interact (90). Research also shows that the food matrix can be impacted if texture enhancers (91, 92) or other additives (93) are included during processing.

#### 3.2.3 Dairy-fat matrix alterations from butter processing

In contrast to milk, butter is a water-in-oil emulsion in which water droplets are suspended in the fat matrix (72, 94). During agitation, or churning, of butter, the MFG is physically disrupted, releasing the TAG core from within the MFGM to aggregate together and form butter. The MFGM fragments coalesce in a separate aqueous buttermilk phase, leaving only a residual MFGM content in butter (33, 36, 94). Though distinctly different from milk, yogurt, and cheese, butter is an important dairy product to consider as it is a concentrated source of dairy fat, presented in a microstructure inverse to that of milk.

As demonstrated, the base product milk can undergo various transformations (i.e., homogenization, heating, coagulation, fermentation, ripening, and churning, respectively) resulting in a variety of dairy products, such as homogenized milk, yogurt, cheese, or butter. These processing methods disrupt or modify the original physico-chemical assembly of dairy lipids in raw milk, resulting in a unique dairy-fat matrix. Here, we evaluate whether, and to what degree, the dairy-fat matrix’s physical (specific packaging and compartmentalization) and chemical assembly (lipid species and other bioactive nutrients) and their interactions influence the bioavailability, digestion, absorption, and metabolism of dairy fat, its moieties, and associated nutrients thereby affecting nutritional and health properties of these dairy products.

## 4 Examining the dairy-fat matrix of different dairy products in cardiometabolic health

Previous reviews and meta-analyses of the literature published prior to 2013 (95–103) represent the early phase of examining the differential effects of individual dairy products on cardiometabolic health. The most consistent finding across these studies was the unique effect of cheese on blood lipids, distinguishing cheese from many other dairy products (95–99). Notably, few of these summarizing works separated dairy intake by both dairy product and by fat content. With these findings, in conjunction with the differences in the dairy-fat matrix across dairy products, we sought to examine if individual dairy-fat matrices have differential effects on cardiometabolic health. Our objective was to utilize a recent snapshot of research (i.e., studies published over the last 10 years) to gain an understanding of the availability of evidence comparing individual dairy-fat matrices (i.e., comparisons stratifying by both dairy product and fat content) and the conclusions we may begin to draw on their unique effects on cardiometabolic health and implications for nutrition guidance.

### 4.1 Search methods

Eligible studies were identified through the Ovid MEDLINE database, using defined search parameters combining the three following categories of search terms with AND: (i) “exp Cholesterol/” OR “exp Dyslipidemias/” OR “Obesity/” OR “Obesity, Abdominal/” OR “exp Hyperglycemia/” OR “exp Insulin Resistance/” OR “Hypertension/” OR “exp Diabetes Mellitus/” OR “Cardiometabolic.mp.” OR “Inflammation/” OR “exp Cardiovascular Diseases/” OR “Stroke/” OR “Non-alcoholic Fatty Liver Disease/” OR “exp Mortality/,” (ii) “exp Dairy Products/,” and (iii) “exp Dietary Fats/.” Two additional searches of Ovid MEDLINE and PubMed^®^ were performed with the keywords “dairy fat.mp.” and [“dairy fat” AND “cardiometabolic health”], respectively. Search results were refined to include studies published from 2013–2023 (searches conducted November 2023) that were written in the English language and performed in humans. Publications were manually excluded from this list if the study: (i) was not a primary research article, (ii) did not have outcome measures related to cardiometabolic health, (iii) used food survey methodology that did not stratify by dairy product or fat content of the dairy product, (iv) compared a component of the dairy product that was not fat, (v) compared dairy products to non-dairy products, (vi) analyzed specific fatty acids as opposed to intake of the whole dairy product, or (vii) focused on non-bovine dairy products (e.g., dairy products made from goat’s milk). Other relevant papers identified throughout the search process were also included. In total, 59 primary research articles were assessed and reported in this review. The terminology for milk-fat content varied greatly among studies, thus, the term “regular-fat,” followed by the respective milk-fat content (if defined), is used to describe what is termed high-fat, full-fat, or whole-fat in the reviewed studies.

### 4.2 Results

We identified 46 observational studies (Supplementary Table S2) and 13 randomized controlled trials (RCTs; Supplementary Table S3) that examined the role of dairy fat consumption on a given cardiometabolic health outcome. In the observational studies, cardiometabolic health outcomes related to obesity, type 2 diabetes (T2D), CVD, inflammation, and the metabolic syndrome (MetS) are presented. Similarly, cardiometabolic health outcomes related to obesity, hyperglycemia and insulin resistance, inflammation, hypertension, and dyslipidemia from the RCTs are presented. The observational studies mainly utilized prospective cohort designs, as opposed to a cross-section design, and the RCTs utilized either a parallel or crossover design.

#### 4.2.1 Observational studies

##### 4.2.1.1 Milk

Thirty-one of the observational studies focused on regular-fat milk intake (Table 1). Their study designs included analyses such as: (i) comparing the effect of one versus three daily servings of regular-fat milk, (ii) comparing the effect of non-fat milk versus regular-fat milk, and (iii) modeling the effect of substituting non-fat milk with regular-fat milk, on a given cardiometabolic health outcome.

**Table 1:** Summary of findings from observational studies (*n* = 31) evaluating the effects of regular-fat milk intake on cardiometabolic disease risk factors and outcomes.

Reference	Dairy product	Obesity	T2D^a^	CVD^b^	Inflammation	MetS^c^
Slurink et al. (104)	Milk	__^d^	↓↔ ^e^	__	__	__
McGovern et al. (105)	Milk	↓ ^f^ ↔	↓↔	↔	↓↔	↔
Slurink et al. (106)	Milk	__	↔	__	__	__
Wang et al. (107)	Milk	__	↔	↑ ^g^ ↔	__	__
Ibsen et al. (108)	Milk	__	↓↔	__	__	__
Cruijsen et al. (109)	Milk	__	__	↔	__	__
Wilkinson et al. (110)	Milk	↑↔	__	__	__	__
Kvist et al. (111)	Milk	__	__	↔	__	__
White et al. (112)	Milk	↓↔	__	__	__	__
Lahoz-García et al. (113)	Milk	↓	__	↓↔	__	__
Ding et al. (114)	Milk	__	__	↑	__	__
Drouin-Chartier et al. (115)	Milk	__	↔	__	__	__
Wong et al. (116)	Milk	__	__	↑	__	__
Kummer et al. (117)	Milk	__	↔	__	__	__
Laursen et al. (118)	Milk	__	__	↔	__	__
Johansson et al. (119)	Milk	__	↔	↑↔	__	__
Koskinen et al. (120)	Milk	__	__	↔	__	__
Johansson et al. (121)	Milk	↓	↓↔	↑↓ ^h^ ↔	__	__
Sun et al. (122)	Milk	__	__	__	__	↔
Brouwer-Brolsma et al. (123)	Milk	__	↑↔	__	__	__
Laursen et al. (124)	Milk	__	__	↔	__	__
Um et al. (125)	Milk	__	__	↔	__	__
Hruby et al. (126)	Milk	__	↑↓↔	__	__	__
Guasch-Ferré et al. (127)	Milk	__	↔	__	__	__
Díaz-López et al. (128)	Milk	__	↔	__	__	__
Ericson et al. (129)	Milk	__	↔	__	__	__
Crichton et al. (130)	Milk	__	__	__	__	↓
Crichton et al. (131)	Milk	↓	__	__	__	__
Avalos et al. (132)	Milk	__	__	↔	__	__
Holmberg et al. (133)	Milk	↓	__	__	__	__
Scharf et al. (134)	Milk	↓↔	__	__	__	__
Patterson et al. (135)	Milk	__	__	↔	__	__

a T2D, type 2 diabetes.

b CVD, cardiovascular diseases.

c MetS, metabolic syndrome.

d Outcome measure(s) not evaluated.

e No disease risk indicated.

f Decreased disease risk indicated.

g Increased disease risk indicated.

h Both increased and decreased disease risk indicated.

Four of the 7 observational studies that investigated the relationship of regular-fat (2–4%) milk intake and obesity were conducted in individuals across childhood stages, utilizing a variety of outcome measures including odds of overweight or obesity as well as associations with specific measurements of adiposity including body mass index (BMI) or waist circumference. Three of these studies reported either no association or an inverse association with the outcome measures evaluated (105, 112, 134) and one reported only inverse associations with the measures of adiposity reported (113). The remaining three studies were conducted in adults; Wilkinson et al. (110) observed no difference in BMI between non-fat and regular-fat (not defined) milk consumers but a 0.6 cm higher sagittal abdominal diameter associated with habitual consumption (i.e., consuming milk ≥5 times per week) of regular-fat milk. Two other studies reported a beneficial relationship between regular-fat (3.0%) milk consumption and outcome measures related to body weight and composition, specifically rates of central obesity and BMI (36 and 48% lower odds of development, respectively), compared to low-fat (≤1.5%) milk consumption (121, 133).

For outcome measures related to prediabetes, one prospective cohort study showed a non-linear association (hazard ratio crosses one at approximately 10 servings of regular-fat milk per week) between regular-fat (not defined) milk intake and prediabetes incidence in a continuous model; however, there was no association found when servings were analyzed categorically (126). Similarly, a different prospective cohort study showed no association between regular-fat (>2%) milk intake and incident prediabetes (106). Brouwer-Brolsma et al. (123) reported a positive association between regular-fat (3.5%) milk consumption and prediabetes risk (7% greater risk) when comparing between the highest and lowest tertiles of regular-fat milk intake, but no association was observed when analyzing per serving increase. Moreover, Slurink et al. (104) observed an inverse association with a relative risk of 0.89 between intake of regular-fat (>2%) milk and prediabetes risk when analyzed in a continuous model, but no association was observed when intake was analyzed in tertiles. Across multiple studies, regular-fat (2.5–4%) milk intake did not affect T2D risk (117, 119, 126–129) or T2D-related mortality (107). Similarly, substituting reduced-fat milk in place of regular-fat (not defined) milk was not associated with risk of T2D (115). Replacing regular-fat (3.5%) milk with skim milk was associated with a 0.4% higher risk of T2D in individuals aged 65–72 years but not in individuals 56–64 years old (108). Two studies focused on specific biomarkers related to T2D risk, including Homeostatic Model Assessment of Insulin Resistance and fasting blood glucose (105, 121). In children, regular-fat (2–3.25%) milk intake compared to low-fat (skim and 1%) milk intake was associated with a lower Homeostatic Model Assessment of Insulin Resistance (*β* coefficient approximately −0.25) in adolescence for participants with a BMI over the 85th age-appropriate percentile, but not participants with a BMI between the 5th and 85th percentiles (105). For adults, an increased regular-fat (3.0%) milk intake was not associated with fasting blood glucose, but when the authors considered adjusted means, regular-fat milk intake was associated with a 0.03 mmol/L lower fasting blood glucose concentration (121).

The majority of the identified observational studies (13 out of 15) that assessed regular-fat (≥3%) milk intake and outcomes related to CVD reported no association with at least one outcome measure (107, 109, 111, 118, 120, 124, 125, 132, 135). These outcomes included CVD-specific mortality (109, 125), myocardial infarction (MI) risk (111, 119, 135), coronary heart disease incidence (120, 132), ischemic heart disease mortality (109), stroke rate (118, 124), or stroke mortality (107, 109). Further, substitution of low-fat (≤1.5%) milk in place of regular-fat (≥3%) milk was not associated with MI risk (135). Three other studies indicated a detrimental effect of regular-fat milk intake and outcomes related to CVD, specifically citing a greater risk of cardiovascular mortality [hazard ratio 1.09 per half a serving increase per day (114)] or stroke incidence [hazard ratio 1.19 when the highest intake quartile was compared to the lowest (119)] associated with intake of regular-fat (3.0%) milk. The third study reported lower risk for heart disease mortality in individuals consuming lower-fat (2% and ≤1%) milk (hazard ratios 0.73 and 0.67, respectively) compared to individuals consuming regular-fat (3–4%) milk (107). Four studies assessed the relationship between regular-fat milk intake and concentrations of blood lipoproteins, such as total CHOL and subclasses of CHOL [e.g., low-density lipoprotein (LDL)-CHOL], but found conflicting results in terms of whether regular-fat milk consumption resulted in a beneficial or detrimental effect on the blood lipid profile (105, 113, 116, 121). Additional studies reported no association between regular-fat (2–3.25%) milk intake and blood pressure (105, 121), and a positive association (correlation coefficient 0.124) between regular-fat (not defined) milk intake and a cardiorespiratory fitness score, indicating improved cardiorespiratory fitness (113).

Only one observational study evaluated outcome measures related to inflammation, and reported a positive association (*β* coefficient approximately 0.25) between adiponectin concentrations and regular-fat (2–3.25%) milk consumption in participants with a BMI over the 85th age-appropriate percentile, but not in participants with a BMI between the 5th and 85th percentile (105).

Two studies utilized calculations to determine the effect of regular-fat milk consumption on general cardiometabolic health. Sun et al. (122) used the continuous MetS risk score, which composites a score based on the International Diabetes Federation risk factors for MetS [i.e., waist circumference, TAG concentration, high-density lipoprotein (HDL)-CHOL concentration, blood pressure, and fasting blood glucose concentration (136)] and found no association between regular-fat (not defined) milk intake and continuous MetS risk score. Another study, using a similar cardiometabolic risk score, also reported no association with this risk score and the consumption of regular-fat (2–3.25%) milk compared to low-fat (1% and skim) milk (105). Crichton et al. (130), alternatively, utilized a cardiovascular health score that compiled information regarding smoking, BMI, physical activity, total CHOL, blood pressure, and fasting blood glucose based on definitions from the American Heart Association, and found that the cardiovascular health score increased by approximately 0.5 points with an increasing intake of regular-fat (not defined) milk, indicating an improvement in cardiovascular health.

Collectively, recent research does not appear to indicate that reducing intake of regular-fat milk is beneficial for cardiometabolic health. Evidence suggests that there is largely no association between regular-fat milk intake and cardiometabolic health, with a potential beneficial effect on risk factors related to obesity, though additional research on the association with obesity and inflammation would bolster the current evidence base.

##### 4.2.1.2 Yogurt

The effect of regular-fat yogurt intake on cardiometabolic health was examined in 12 recent observational studies (Table 2). Increasing intake of regular-fat (not defined) yogurt was associated with improvements in multiple facets of body composition, including 15–42% lower odds of developing abdominal (131, 140) or generalized (131) obesity. Additionally, though a larger decrease in waist circumference (absolute yearly decrease of 0.27 cm versus 0.15 cm, respectively), was observed among individuals in the highest quintile of regular-fat (not defined) yogurt consumption compared to the lowest quintile of consumption this decrease was not large enough to constitute a decrease in abdominal obesity in these same individuals (139). Another study indicated no association between increased regular-fat (≥3.9%) yogurt consumption and body weight, BMI, waist circumference, or waist to hip ratio (138).

**Table 2:** Summary of findings from observational studies (*n* = 12) evaluating the effects of regular-fat yogurt intake on cardiometabolic disease risk factors and outcomes.

Reference	Dairy product	Obesity	T2D^a^	CVD^b^	Inflammation	MetS^c^
Machlik et al. (137)	Yogurt	__	__	↔	__	__
Ibsen et al. (108)	Yogurt	__^d^	↔ ^e^	__	__	__
Kvist et al. (111)	Yogurt	__	__	↔	__	__
Trichia et al. (138)	Yogurt	↔	↔	↔	__	↔
Laursen et al. (118)	Yogurt	__	__	↓ ^f^ ↔	__	__
Brouwer-Brolsma et al. (123)	Yogurt	__	↑ ^g^ ↔	__	__	__
Guasch-Ferré et al. (127)	Yogurt	__	↓	__	__	__
Santiago et al. (139)	Yogurt	↓↔	__	__	__	__
Díaz-López et al. (128)	Yogurt	__	↓	__	__	__
Sayón-Orea et al. (140)	Yogurt	↓	↔	↔	__	↔
Crichton et al. (130)	Yogurt	__	__	__	__	↔
Crichton et al. (131)	Yogurt	↓	__	__	__	__

a T2D, type 2 diabetes.

b CVD, cardiovascular diseases.

c MetS, metabolic syndrome.

d Outcome measure(s) not evaluated.

e No disease risk indicated.

f Decreased disease risk indicated.

g Increased disease risk indicated.

Brouwer-Brolsma et al. (123) observed a 7% greater risk of prediabetes associated with regular-fat (2.9%) yogurt consumption when comparing between the highest and lowest tertiles of intake (no association was observed, though, when analyzed per serving increase); however, no association was observed between regular-fat yogurt consumption and T2D risk in the same individuals. Three other prospective cohort studies reported no relationship between regular-fat (≥3.5%) yogurt consumption and T2D risk (108) or T2D risk factors of fasting blood glucose concentration (140) or hemoglobin A1c percentage (138). Moreover, two prospective cohort studies reported a 34–35% lower risk of T2D associated with regular-fat (not defined) yogurt consumption (127, 128).

Related to CVD outcomes, replacing low-fat (<2%) yogurt with regular-fat (≥3%) yogurt was not associated with risk of MI (111), total stroke (118), or hemorrhagic stroke (118); this substitution, though, was associated with a greater rate of ischemic stroke [hazard ratio 2.58 with one serving substitution per day (118)]. Three other studies reported no association between greater consumption of regular-fat (≥3.9%) yogurt and changes in blood lipid concentrations (TAG, HDL-CHOL, LDL-CHOL, or total CHOL), or blood pressure, indicators of cardiovascular health (137, 138, 140).

Sayón-Orea et al. (140) and Crichton et al. (130) evaluated the effect on regular-fat (not defined) yogurt consumption on the risk of MetS (i.e., according to the criteria set forth by the American Heart Association and the International Diabetes Federation) and cardiovascular health score, respectively; neither found regular-fat yogurt consumption to be associated with these scores. Trichia et al. (138) used a similar metabolic risk score and also reported no association between increasing regular-fat (≥3.9%) yogurt and the metabolic risk score.

In summary, the observational studies discussed above report largely no association between regular-fat yogurt consumption and cardiometabolic health. Consumption of regular-fat yogurt appears specifically effective in protecting against obesity; a weaker effect was observed on outcome measures related to T2D or CVD.

##### 4.2.1.3 Cheese

Out of the 14 recent studies that assessed the relationship between regular-fat cheese consumption and cardiometabolic health, only three studies investigated the relationship between intake of regular-fat cheese and obesity (Table 3). One study reported no association between regular-fat (not defined) cheese consumption and both abdominal and generalized obesity (131). Trichia et al. (138) reported no association with regular-fat (≥3.9%) cheese intake and waist circumference or waist to hip ratio. These authors also found a positive association between regular-fat (≥3.9%) cheese intake and change in body weight (approximately 0.3 kg) as well as BMI (approximately 0.25 kg/m^2^) in participants ages 50–60 years but no association was found in participants aged 40–50 or 60–78 years (138). In the third study, participants that consumed regular-fat (≥28%) cheese, compared to lower-fat (10–17%) cheese, had 13% lower odds of overweight or obesity (121).

**Table 3:** Summary of findings from observational studies (*n* = 14) evaluating the effects of regular-fat cheese intake on cardiometabolic disease risk factors and outcomes.

Reference	Dairy product	Obesity	T2D^a^	CVD^b^	Inflammation	MetS^c^
Slurink et al. (104)	Cheese	__^d^	↔ ^e^	__	__	__
Machlik et al. (137)	Cheese	__	__	↑↓ ^f^ ↔	__	__
Slurink et al. (106)	Cheese	__	↓ ^g^	__	__	__
Shi et al. (141)	Cheese	__	↓↔	↓↔	↓↔	__
Trichia et al. (138)	Cheese	↑ ^h^ ↔	↔	↑↓↔	__	↔
Johansson et al. (119)	Cheese	__	↔	↔	__	__
Koskinen et al. (120)	Cheese	__	__	↔	__	__
Johansson et al. (121)	Cheese	↓	↑↓	↑↓↔	__	__
Brouwer-Brolsma et al. (123)	Cheese	__	↔	__	__	__
Karatzi et al. (142)	Cheese	__	__	↔	__	__
Brouwer-Brolsma et al. (143)	Cheese	__	↔	__	__	__
Crichton et al. (130)	Cheese	__	__	__	__	↔
Crichton et al. (131)	Cheese	↔	__	__	__	__
Patterson et al. (135)	Cheese	__	__	↓↔	__	__

a T2D, type 2 diabetes.

b CVD, cardiovascular diseases.

c MetS, metabolic syndrome.

d Outcome measure(s) not evaluated.

e No disease risk indicated.

f Both increased and decreased disease risk indicated.

g Decreased disease risk indicated.

h Increased disease risk indicated.

For outcome measures related to prediabetes, Brouwer-Brolsma et al. (123) and Slurink et al. (104) observed no association between regular-fat (>20%) cheese consumption and prediabetes incidence, while Slurink et al. (106) found that a greater intake of regular-fat (>20%) cheese was associated with a 22% lower risk of prediabetes incidence. Three studies reported no association between regular-fat (≥24%) cheese consumption and T2D incidence (119, 123, 143). The results regarding the relationship between regular-fat cheese intake and the risk factors related to prediabetes and T2D are inconclusive; these include, but are not limited to (i) evidence of a beneficial relationship [increasing regular-fat (not defined) cheese intake was associated with a 0.2% lower circulating glucose concentrations (141)], (ii) evidence of an unfavorable relationship [consumers of regular-fat (≥28%) cheese had a 7% greater odds of a fasting blood glucose concentration in the prediabetic range (121)], and (iii) evidence of no relationship [increasing intake of regular-fat (≥3.9%) cheese was not associated with hemoglobin A1c concentration (138)].

In other recent work, regular-fat (≥3.5%) cheese consumption was not associated with coronary heart disease incidence (120), stroke incidence (119), or retinal vessel calibers [biomarker of CVD risk (142)]. In a cohort of Swedish women, substituting low-fat (10–17%) cheese in place of regular-fat (<17%) cheese did not affect MI risk, but increasing intake of regular-fat cheese was associated with a 17% lower MI risk (135). A study in another cohort of Swedish women and men reported no association between regular-fat (≥28%) cheese intake and MI risk (119). Four observational studies evaluated the association between regular-fat (≥3.9%) cheese intake and specific CVD risk factors, including blood pressure, lipids, and lipoproteins (121, 137, 138, 141). HDL-CHOL and LDL-CHOL concentrations were either not associated or positively associated with regular-fat (≥3.0%) cheese intake, while blood pressure and TAG concentrations were either not associated or inversely associated with regular-fat (≥3.9%) cheese intake (121, 137, 138, 141). The relationship between regular-fat (≥3.9%) cheese consumption and total CHOL concentration was less clear as there were studies that reported no association (137, 141), a positive association (138), or a combination of associations between these two variables based on different analytical stratifications and adjustments (121).

Related to inflammation, one study reported an inverse association between regular-fat (not defined) cheese intake and C-reactive protein and interleukin (IL)-6 concentrations (3.4 and 3% lower concentrations, respectively), but no association with IL-10, adiponectin, tumor necrosis factor α (TNFα), or TNFα receptor concentrations (141). Two studies reported no association between regular-fat (≥3.9%) cheese intake and cardiovascular health score (130) or metabolic risk score (138), as detailed above.

Collectively, these recent observational studies largely suggest no effect of regular-fat cheese consumption on cardiometabolic health outcomes, with some studies proposing a beneficial effect on outcome measures related to T2D and CVD.

##### 4.2.1.4 Butter

Of the 17 identified studies that surveyed butter consumption, only two evaluated the effect of butter consumption on measures of body weight and composition (Table 4). Trichia et al. (138) found no association between butter intake and body weight, BMI, waist circumference or waist to hip ratio. Johansson et al. (121) found that increasing butter intake was associated with a 28–40% lower odds of overweight or obesity in a cross-sectional analysis, but statistical significance was attenuated in their prospective analysis.

**Table 4:** Summary of findings from observational studies (*n* = 17) evaluating the effects of butter intake on cardiometabolic disease risk factors and outcomes.

Reference	Dairy product	Obesity	T2D^a^	CVD^b^	Inflammation	MetS^c^
Van Parys et al. (144)	Butter	__^d^	__	↔ ^e^	__	__
Shi et al. (141)	Butter	__	↑↔	↑↓ ^f^	↔	__
Cruijsen et al. (109)	Butter	__	__	↔	__	__
Zhang et al. (145)	Butter	__	__	↑ ^g^ ↔	__	__
Trichia et al. (138)	Butter	↔	↔	↑↔	__	↔
Johansson et al. (119)	Butter	__	↔	↔	__	__
Koskinen et al. (120)	Butter	__	__	↔	__	__
Johansson et al. (121)	Butter	↓ ^h^ ↔	↑↓↔	↑↓↔	__	__
Dehghan et al. (146)	Butter	__	__	↔	__	__
Guasch-Ferré et al. (127)	Butter	__	↑	__	__	__
Drehmer et al. (147)	Butter	__	__	__	__	↓
Brouwer-Brolsma et al. (143)	Butter	__	↔	__	__	__
Hosseinpour-Niazi et al. (148)	Butter	__	__	↑	__	↑
Drehmer et al. (149)	Butter	__	↔	__	__	__
Buijsse et al. (150)	Butter	__	↔	__	__	__
Crichton et al. (130)	Butter	__	__	__	__	↔
Avalos et al. (132)	Butter	__	__	↔	__	__

a T2D, type 2 diabetes.

b CVD, cardiovascular diseases.

c MetS, metabolic syndrome.

d Outcome measure(s) not evaluated.

e No disease risk indicated.

f Both increased and decreased disease risk indicated.

g Increased disease risk indicated.

h Decreased disease risk indicated.

Three studies published over the last 10 years reported no association between butter intake and T2D incidence (119, 143, 150); however, one study observed an increased risk of T2D when butter consumption increased [hazard ratio 2.42 with a one serving increase (127)]. In addition to T2D incidence, four studies utilized a variety of biomarkers to evaluate T2D risk, including, but not limited to, blood glucose and insulin concentrations as well as hemoglobin A1c percentage (121, 138, 141, 149). Drehmer et al. (149) found no association between butter intake and fasting blood glucose or insulin concentrations or the concentrations of blood glucose or insulin 2 hours after an oral glucose tolerance test. Shi et al. (141) identified a positive association between butter intake and circulating insulin concentrations (2.3% higher concentrations), but no association with circulating glucose concentrations, while in a cross-sectional analysis, stratified by sex, an increased butter intake was reported to be inversely associated with fasting blood glucose concentrations in male participants (12% lower odds of developing a fasting blood glucose ≥6.1 mmol/L), but was not associated with fasting blood glucose concentrations in female participants (121). Moreover, Johansson et al. (121) ascertained in their prospective analysis that an increased butter intake was positively associated with fasting blood glucose concentrations (28% higher risk of developing a fasting blood glucose ≥6.1 mmol/L). In addition, two studies reported no association between butter intake and hemoglobin A1c percentage (138, 149).

Results related to CVD are also mixed due to the variety of outcome measures utilized. Butter intake was not associated with major cardiovascular events (119, 144, 146), incident coronary heart disease (120, 132), stroke rate or stroke mortality (109, 144, 145), CVD mortality (109, 144), or ischemic heart disease mortality (109), but one study did report that greater butter intake was associated with an 8% greater risk of both CVD and heart disease mortality (145). In terms of CVD risk factors, butter intake was associated with increased LDL-CHOL and total CHOL concentrations, but the relationship with HDL-CHOL and TAG concentrations is less clear as there were studies that reported no association, a positive association, and an inverse association between butter intake and these biomarkers (121, 138, 141, 148).

Only one study evaluated the relationship between butter consumption and inflammation, and proposed no relationship, as evidenced by the lack of association with C-reactive protein, IL-6, IL-10, TNFα, or TNFα receptor concentrations (141).

Four studies analyzed the relationship between butter consumption and broader indices of cardiometabolic health, noting different relationships. Drehmer et al. (147) used a metabolic risk score (i.e., a compilation of the following metabolic risk factors: waist circumference, TAG concentration, HDL-CHOL concentration, systolic blood pressure, and fasting blood glucose concentration) and observed a 0.129-point decrease in the metabolic risk score associated with increasing butter consumption by one serving, suggesting a lower risk of MetS. Hosseinpour-Niazi et al. (148) observed 2.03 times greater odds of MetS [using the globally harmonized criteria (151)] associated with greater butter intake. Finally, Crichton et al. (130) and Trichia et al. (138) observed no association between butter consumption and cardiovascular health score, as described above, or a metabolic risk score, respectively.

Overall, the observational studies report mainly no effect of butter consumption on cardiometabolic health with fewer studies (i.e., 5 of 17 studies) reporting an adverse effect. When evaluating disease outcomes, most studies reported no association with butter consumption, but when evaluating risk factors for these outcomes, there is more evidence to suggest an adverse effect, highlighting a key area for future research. Notably, there was only one component of cardiometabolic health, blood TAG concentration, in which butter consumption appeared to be beneficial, yet this finding was not consistent across all studies that measured this marker.

##### 4.2.1.5 Comparisons between dairy products

In addition to the aforementioned studies that evaluated the impact of dairy fat on cardiometabolic health by dairy product, three recent prospective cohort studies modeled the effects of substituting different regular-fat dairy products for one another on cardiometabolic health outcomes (Table 5; Supplementary Table S2). First, the consumption of regular-fat (3.5%) yogurt, as a substitute for regular-fat (3.5%) milk, was reported to be associated with a 0.7% reduced risk of T2D in individuals 56–59 years old per 50 g per day substitution, but not in adults 60–64 or 65–72 years old (108). Another study showed that regular-fat (3.5%) yogurt substitution in place of regular-fat (3.5%) milk was associated with a 13% lower risk of MI per 200 g per day substitution (111). In modeling how the substitution of several different dairy products affect the rate of total stroke and stroke subtypes, the substitutions of neither regular-fat (≥3%) milk nor regular-fat (≥3%) yogurt, in place of butter, were associated with a change in rate of total or hemorrhagic stroke (118). Regular-fat (≥3%) yogurt as a substitute for butter, however, was associated with a 61% lower rate of ischemic stroke per serving substitution per day (118). Moreover, regular-fat (≥3%) yogurt, as a substitute for regular-fat (≥3%) milk, was associated with a 65% lower rate of ischemic stroke per substitution per day, but not total or hemorrhagic stroke (118).

**Table 5:** Summary of findings from observational studies (*n* = 3) comparing the effect of the substitution between dairy products (milk, yogurt, cheese, or butter) on cardiometabolic disease outcomes.

Reference	Substitution	T2D^a^	CVD^b^
Laursen et al. (118)	Milk as a substitute for butter	__^c^	↔ ^d^
Ibsen et al. (108)	Yogurt (3.5%) as a substitute for milk (3.5%)	↓ ^e^ ↔	__
Kvist et al. (111)	Yogurt as a substitute for milk	__	↓
Laursen et al. (118)	Yogurt as a substitute for milk	__	↓↔
Laursen et al. (118)	Yogurt as a substitute for butter	__	↓↔

a T2D, type 2 diabetes.

b CVD, cardiovascular diseases.

c Outcome measure(s) not evaluated.

d No change in outcome measure(s).

e Decreased disease risk indicated.

In summary, these modeling-based observational studies infer that dairy fat consumed in yogurt may be beneficial for reduction of T2D and CVD risk.

#### 4.2.2 Randomized controlled trials

##### 4.2.2.1 Comparisons within dairy products

Out of the 13 RCTs identified, 6 RCTs evaluated the effect of consuming differing amounts of dairy fat within the same dairy product (e.g., reduced-fat versus regular-fat cheese consumption; Table 6; Supplementary Table S3). Of these, three RCTs focused specifically on milk. There was no effect of regular-fat milk intake observed for many aspects of cardiometabolic health [i.e., there was no association with outcome measures related to obesity (152, 156), hyperglycemia and insulin resistance (152, 156), inflammation (155), or blood pressure (156)]. For outcome measures related to dyslipidemia, largely no effect was found (156), but there were certain outcome measures that were affected by regular-fat milk intake (152, 155). In a 3 week trial with 17 adults, daily consumption of 500 mL (~2 cups) of regular-fat (3.5%) milk, compared to skim (0.1%) milk, did not affect blood total CHOL, LDL-CHOL, or TAG concentrations, but resulted in a 0.06 mmol/L increase in HDL-CHOL concentrations (152). Similarly, another study found that HDL-CHOL concentrations were 0.16 mmol/L higher after the daily consumption of 400 mL of regular-fat (3%) milk compared to skim (0.5%) milk in children, over a 4 month period, along with total CHOL, LDL-CHOL, and total apolipoprotein (Apo)-B concentrations (0.43 mmol/L, 0.28 mmol/L, and 0.05 g/L greater, respectively) as well as the total Apo-B:Apo-A1 ratio (0.05 g/L greater) (155). This study also reported no change in blood TAG, very low-density lipoprotein CHOL, or Apo-A1 concentrations, as well as no change in the total CHOL:HDL-CHOL ratio (155).

**Table 6:** Summary of findings from randomized controlled trials (*n* = 6) comparing the effects of individual dairy products (milk, cheese, or butter) on cardiometabolic risk factors.

Reference	Comparison	Obesity	Hyperglycemia and insulin resistance	Inflammation	Hypertension	Dyslipidemia
Engel et al. (152)	Whole milk (3.5% fat) versus skim milk (0.1% fat)	↔ ^a^	↔	__^b^	__	↓ ^c^ ↔
Drouin-Chartier et al. (153)	Cheddar cheese (32% fat) versus cream cheese (31% fat)	__	__	__	__	↑ ^d^ ↔
Raziani et al. (154)	High-fat cheese (25%/32% fat) versus reduced fat cheese (13%/16% fat)	↔	↔	↔	__	↔
Villalpando et al. (155)	Whole milk (3% fat) versus skim milk (0.5% fat)	__	__	↔	__	↑↓ ^e^ ↔
Loria-Kohen et al. (156)	Semi-skim milk (~1.9% fat) versus skim milk (~0.3% fat)	↔	↔	__	↔	↔
Penedo et al. (157)	No dairy fat consumption versus habitual dairy fat consumption with an additional 20 g/d butter	↔	↔	↓↔	__	__

a No change in outcome measure(s).

b Outcome measure(s) not evaluated.

c Decreased disease risk indicated.

d Increased disease risk indicated.

e Both increased and decreased disease risk.

The remaining three studies compared dairy fat intake within either cheese or butter utilizing different study designs. For example, a meal tolerance test comparing 33 g of fat from either cream cheese or cheddar cheese resulted in no differences in the area under the curve for TAG or free FA concentration change compared to baseline, but cheddar cheese consumption resulted in a larger area under the curve for Apo-B48 concentrations (60,237 ng/mL/h following cheddar cheese consumption compared to 58,528 ng/mL/h following cream cheese consumption) (153). Alternatively, no changes were observed in a 12 week RCT assessing the effect of daily consumption of regular-fat (25–32%) versus reduced-fat (13–16%) cheese on markers of body weight, blood glucose control, blood lipid profile, or inflammation (154). In a depletion-repletion study where dairy fat consumption was first eliminated for 8 weeks, then permitted with the addition of 20 g per day of butter for the subsequent 8 weeks, there were also no changes in BMI, body fat mass, or fasting blood glucose (157). Additionally, this study reported no change in C-reactive protein, adiponectin, or serum IL-4 concentrations after the repletion period, but an improvement in many other inflammatory markers [e.g., a decrease in TNFα concentration (157)].

Collectively, the limited evidence from these RCTs suggests that the consumption of regular-fat milk, cheese, or butter compared to lower-fat options may not affect cardiometabolic disease risk factors.

##### 4.2.2.2 Comparisons between dairy products

Eight RCTs were identified that compared the effects of dairy fat consumed across different dairy products on biomarkers of cardiometabolic health (Table 7; Supplementary Table S3). Seven of the 8 RCTs compared the effects of cheese intake with either butter or milk intake; these studies mostly found that the consumption of these regular-fat dairy products did not differentially affect body weight, body composition, hyperglycemia and insulin resistance, hypertension, or inflammation (160, 163, 164). In contrast, reported effects of dairy fat consumption on markers of dyslipidemia were more variable among different types of dairy products (160–164). As an example, Rancourt-Bouchard et al. (164) identified that the consumption of regular-fat (31%) cheese enriched with γ-aminobutyric acid, compared to low-fat (1%) milk, increased total-CHOL, LDL-CHOL, and Apo-B100 concentrations (by 0.23 mmol/L, 0.21 mmol/L, and 0.05 g/L, respectively), but also increased LDL-CHOL size by 0.6 Å and Apo-A1 concentration by 0.05 g. However, there were no diet-related changes in the total CHOL to HDL-CHOL ratio, HDL-CHOL concentration, or TAG concentration in response to consuming the diet with regular-fat cheese enriched with γ-aminobutyric acid compared to the diet with low-fat milk (164). Feeney et al. (160) compared the effect of a diet containing 40 g of dairy fat consumed daily as either (i) regular-fat cheese or (ii) reduced-fat cheese and butter, for 6 weeks on cardiometabolic health markers. Greater decreases in total CHOL concentrations (0.15 mmol/L greater decrease) and LDL-CHOL concentrations (0.18 mmol/L greater concentrations), but not HDL-CHOL, TAG, or free FA concentrations, were observed in response to the regular-fat cheese diet (160). No diet-related differences were observed in fasting blood glucose or insulin concentrations (160). In a secondary analysis, no differences were seen in additional markers of hyperglycemia and insulin resistance (i.e., lipoprotein insulin resistance score) or dyslipidemia [i.e., LDL-CHOL, HDL-CHOL, very LDL-CHOL/chylomicron, and intermediate-density lipoprotein concentration or size (159)]. However, one study that did not report any differences in the blood lipid profile was a meal tolerance test that compared the consumption of 33 g of fat from either cream cheese, cheddar cheese, or butter; there were no differences in TAG, Apo-B48, or free FA concentrations between the consumption of butter and either type of cheese (153).

**Table 7:** Summary of findings from randomized controlled trials (*n* = 9) comparing the effects of substitution between dairy products (milk, yogurt, cheese, or butter) on cardiometabolic disease risk factors.

Reference	Comparison	Obesity	Hyperglycemia and insulin resistance	Inflammation	Hypertension	Dyslipidemia
Chen et al. (158)	Yogurt (2.9% fat) versus milk (3.7% fat)	↓ ^a^	↓↔ ^b^	↓↔	↔	↓↔
Dunne et al. (159)	Cheese (34% fat) versus cheese and butter (~31% fat)	__^c^	↔	↔	__	↔
Feeney et al. (160)	Cheese (34% fat) versus cheese and butter (~31% fat)	↔	↔	↔	↔	↓↔
Drouin-Chartier et al. (153)	Cheddar cheese (32% fat) versus butter	__	__	__	__	↔
Drouin-Chartier et al. (153)	Cream cheese (31% fat) versus butter	__	__	__	__	↔
Rancourt-Bouchard et al. 20,202 (159)	Cheese (31% fat) versus low-fat milk (1% fat)	↔	↔	↔	↔	↑↓ ^d^ ↔
Brassard et al. (161)	Cheese versus butter	__	__	__	__	↓↔
Hansson et al. (162)	Cheese (38% fat) versus butter (82% fat)	__	↑ ^e^ ↔	__	__	↔
Brassard et al. (163)	Cheese versus butter	↔	↔	↔	↔	↑↓↔

a Decreased disease risk indicated.

b No change in outcome measure(s).

c Outcome measure(s) not evaluated.

d Both increased and decreased disease risk indicated.

e Increased disease risk indicated.

The eighth RCT compared daily intake of 220 g of either regular-fat (2.9%) yogurt or regular-fat (3.7%) milk in Chinese women with an elevated waist circumference and BMI (158). After 24 weeks of regular-fat yogurt consumption, the authors observed either no change or an improvement in outcome measures related to obesity and body composition, hyperglycemia and insulin resistance, inflammation, hypertension, and dyslipidemia.

In summary, these few RCTs primarily indicate no effect of dairy fat consumption on cardiometabolic risk factors when comparing intake of dairy fat in cheese to that in butter or milk. More clinical research is needed however, particularly on lipid homeostasis outcome measures as well as research including other dairy products, particularly yogurt.

### 4.3 Discussion

#### 4.3.1 Findings, potential biological mechanisms, and methodological considerations

Limiting dairy fat intake is a prominent dietary recommendation to reduce energy and SFA intake to lower cardiometabolic disease risk. However, this recommendation is derived in part from a single-nutrient approach to dietary guidance, which does not fully recognize the importance of the food matrix or the dairy-fat matrix. As a result of the different dairy-fat matrices evident in milk, yogurt, cheese, and butter, our aim was to identify and evaluate observational studies and RCTs conducted within the previous 10 years that focused on the effect of the different dairy products on cardiometabolic health. There was primarily no effect observed of the consumption of these individual regular-fat dairy products on cardiometabolic health, as evidenced by both observational studies and RCTs; however, the variable nature of the observed results (Supplementary Tables S4–S6), highlight the need for additional research. Results from observational studies suggest that regular-fat milk intake may have a beneficial effect on risk factors related to obesity, RCTs however, did not report an effect. Observational studies with results on the cardiometabolic health effect of regular-fat yogurt consumption identified a more substantial beneficial effect on obesity and body weight regulation, while the results from the analyses of substitutions between dairy products demonstrate a potentially beneficial effect of regular-fat yogurt intake on T2D and CVD risk. Only one recent RCT is available to inform the limited scope of evidence on regular-fat yogurt consumption and aligns with the observational findings. Of note, the U.S. Food and Drug Administration recently announced a qualified health claim for yogurt’s potential role in reducing risk of T2D (165), and this claim was not limited to yogurts of a specific fat content (166). Results from both study types noted that consumption of regular-fat cheese may affect the blood lipid profile, in alignment with the initial work published prior to 2013 examining the differential effects of individual dairy products on cardiometabolic health, though the directionality of this effect is not definitive and requires further research. In the observational studies, results for butter were not conclusive and the reviewed studies showcased either no effect, an increased effect, or a decreased effect on cardiometabolic disease risk, while most RCTs demonstrated primarily no effect of butter consumption on cardiometabolic disease risk factors. As shown in Supplementary Table S6, 63% of the conclusions drawn from the reviewed studies indicate no effect of dairy-fat intake on cardiometabolic health outcomes, while 24 and 13% of the conclusions drawn indicate an increase or decrease, respectively, in cardiometabolic disease risk. Together, these heterogeneous results, stratified by dairy product and fat content suggest primarily no effect of consuming higher-fat varieties of dairy products on cardiometabolic health, indicating that it likely may not be necessary to limit dairy fat intake within an overall healthy, energy-balanced diet.

Many reviews have thoroughly examined the effects of the unique physico-chemical assembly of dairy products on digestion and absorption (4, 30, 71, 72, 167), with notable mechanisms including, but not limited to, the effect of MFG size on lipase action and intestinal absorption as well as the effect of calcium content on dietary fat absorption due to the interplay of calcium and FAs, and their ability to form calcium soaps, highlighting the importance of the other macro- and micronutrients present in the matrix of the dairy product. In fact, the presence of these other macro- and micronutrients in dairy products may contribute to the observed differences in health effects between dairy-derived SFAs and SFAs from other food sources (167–169). For example, the higher calcium content in cheese may be relevant to the potential effect of regular-fat cheese intake on blood lipid control (30). Moreover, specific dairy bioactives may elicit cardiometabolic health effects such as the MFGM components and their hypothesized effect on aspects of cardiometabolic health including, but not limited to, CHOL absorption and inflammation (4, 8, 41, 152, 170–173). Further, the small MFGs found in homogenized milk both on the market and homogenized milk used as a base product for yogurt (i.e., compared to hard cheeses that are typically made from unhomogenized milk) may impact satiety and lipid bioavailability (4, 30, 71), which may be important for the absorption of the MCFAs that have been thought to help with body-weight control (30, 170). The ability of specific dairy components to drive cardiometabolic health effects is dependent on their bioavailability, which is impacted by the food and fat matrix of each dairy product.

**Figure 3:**
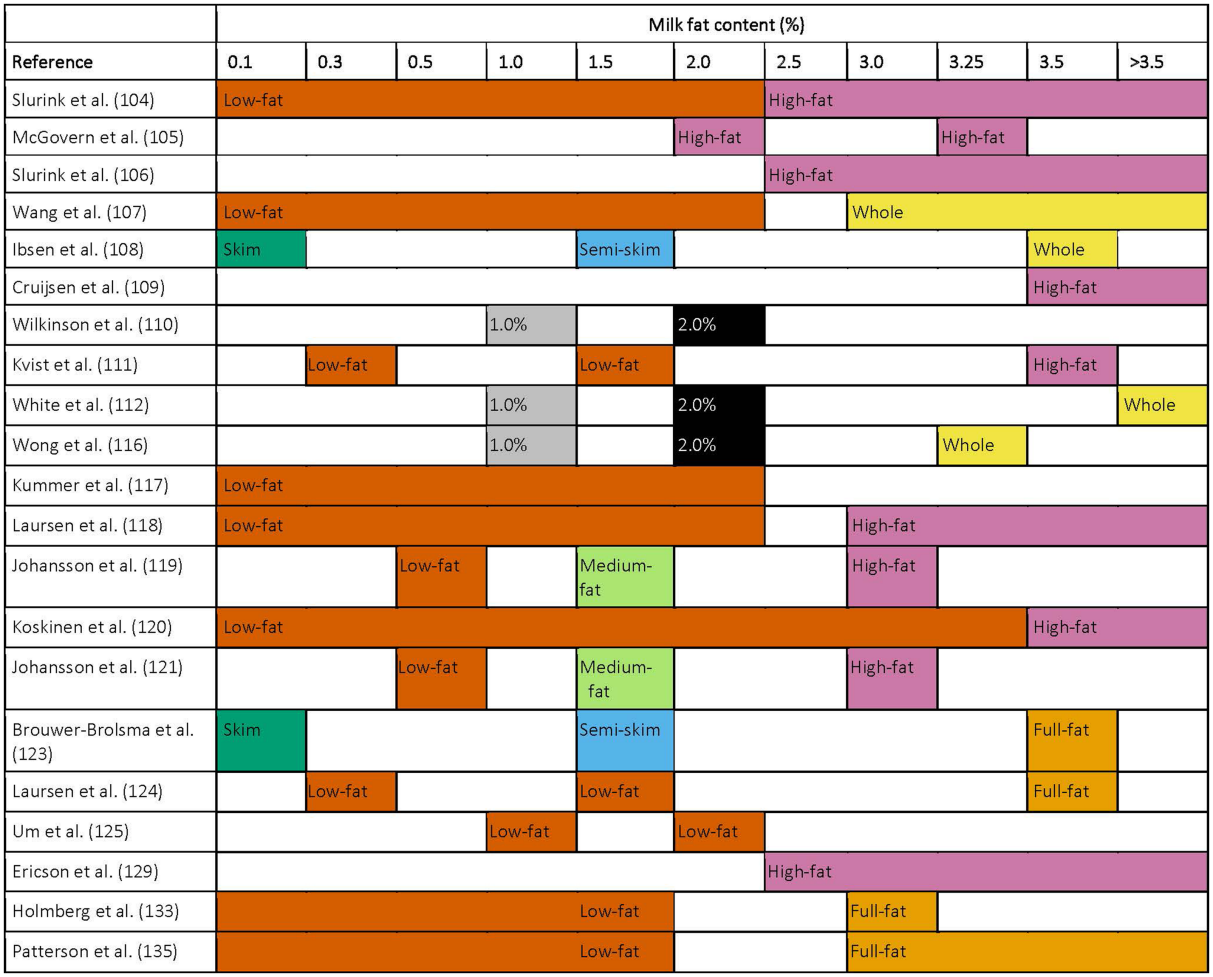
Milk-fat-content definitions used in observational studies with terminology defined.

There are many nutritional, lifestyle, and methodological factors at play in the studies reviewed that ought to be considered for the existing body of literature and for future research in this area. For example, dietary patterns may have a modulatory effect of specific dairy products on cardiometabolic health, thus, an important consideration in this area of research is the diet in which these dairy products are eaten (174). A confounding mechanism may be the subsequent effect of either adding or removing dietary fat in general: e.g., an increase in caloric intake corresponding with the inclusion of dairy fat (110, 123) or a decrease in carbohydrate consumption that corresponds with an increase in dietary fat (160, 175). In these cases, it is difficult to distinguish whether the cardiometabolic health outcomes are due to an increase in overall fat consumption or rather a difference in total energy intake or distribution. A limitation across both the observational studies and RCTs identified is the lack of detail and standardization in dietary intake methodology (e.g., in the collection and reporting of dairy fat intake). In the observational studies reviewed, four dietary intake methods were described: (i) food recalls, including 24 h recalls (*n* = 4 studies), (ii) food records (*n* = 1 study), (iii) food frequency questionnaires (FFQs; *n* = 39 studies), or (iv) the Diet Behavior and Nutrition Survey from the National Health and Nutrition Examination Survey (*n* = 2 studies). Though most studies used an FFQ, the FFQs varied considerably in the number of total items or questions, ranging from a dairy-specific 15-question FFQ to a more generalized 261-question FFQ. Additionally, milk-fat content terminology is generally not standardized and is largely variable, mainly in milk with less than 2% fat content (Figure 3). Some authors use the terms “skim” or “semi-skimmed” milk, while others state the milk fat percentages. In contrast, other studies broadly define a “low-fat” milk category, including fat contents below a certain threshold, such as 2%. This terminology often creates a greater variance within this “low-fat” group than the variance between the “low-fat” and regular-fat groups. For example, there could be a difference of up to 2% between non-fat and 2% milk, compared to a difference of 1.25–1.5% between the “low-fat” and regular-fat groups. Differences in survey methods may impact the quality of the data being collected, as all methods have strengths and weaknesses (176). Similarly, differences in milk-fat content definitions greatly impact generalizability, as different authors may use different terms for the same milk-fat content or may refer to different milk-fat contents using the same term. Clear definition and standardization of dairy fat intake data collection and reporting would allow for comparison between studies and could yield more robust and cohesive research within the field.

Another limitation is the variability of study designs utilized. Across the observational studies, there were three types of analyses used: (i) comparisons between high and low intake of a regular-fat dairy product (e.g., quartiles of regular-milk intake), (ii) comparisons between intake of a low-fat dairy product with a regular-fat dairy product (e.g., skim milk compared to regular-fat milk), or (iii) substitution analyses in which a dairy product of a different type or fat content was substituted for another regular-fat dairy product (e.g., low-fat milk substituted for regular-fat milk). Similarly, the RCTs utilized different degrees of control, including: (i) providing only a controlled food item (i.e., a specific serving of the dairy product of interest), (ii) recommending a lower-fat meal prior to post-prandial experiments, (iii) limiting dairy consumption by amount or by dairy product, or (iv) restricting all dairy products beyond the dairy product of interest. In addition, many different control groups were used, with studies including comparisons of a high-fat dairy product to either a: (i) low-fat dairy product of the same type, (ii) low-fat dairy product of a different type, (iii) high-fat dairy product of a different type, and/or (iv) non-dairy control as well as a depletion/repletion design. The variety in study design provides many avenues to examine the effect of the dairy-fat matrix in cardiometabolic health outcomes and explores different dietary changes likely to be employed by consumers (e.g., increasing intake of a food or substituting it for another). Yet, the differences also impart barriers in comparing results across studies.

#### 4.3.2 Strengths, weaknesses, and future directions

Our focus on research published within the last 10 years yields a timely review of the literature and provides an informative snapshot of emerging research to better understand the role of the dairy-fat matrix on cardiometabolic health. This narrative review evaluates the individual effects of cow’s milk, yogurt, cheese, and butter, but does not evaluate the effect of total dairy product consumption or combinations of dairy products (i.e., the consumption of different dairy products eaten within a meal or over the course of one day). Further, without evaluating the effect of total dairy fat consumption, this review does not allow for evaluation of recommendations for frequency or quantity of total dairy fat intake but is an important area of future research. Finally, with the focus on commonly consumed dairy products, this review does not consider the full range of dairy products available on the market (e.g., fermented milk or ice cream), but highlights many opportunities for future research.

The literature currently skews toward nonfermented dairy products (i.e., milk and butter) over fermented dairy products (i.e., yogurt and cheese), with only one recently conducted RCT including yogurt. Yogurt and other fermented dairy products are important topics of future research as recent observational studies suggest cardiometabolic benefits of regular-fat fermented dairy product intake (106, 129).

Diversity in study cohorts is crucial as there are disparities in cardiometabolic risk factors and outcomes by sex, age, education, race, and ethnicity that deserve recognition and future research funding and exploration (177). Most studies recruited a cohort of mostly equal numbers (less than 10% difference) of male and female participants. The average age of participants in the observational studies appeared to be split approximately evenly between the ranges of 30–50 years, 50–60 years, and 60–70 years old, while the average age of RCT participants was mostly under 50 years old. Notably, there were few studies that recruited children, and they all only evaluated the relationship between cardiometabolic health and regular-fat milk. Additionally, while a little over half of the observational studies recruited participants older than 70 years old, this was only the case for one out of the 13 RCTs. Less information is available for the educational status of the participants studied. This information was not reported in approximately a third of the observational studies and not reported in any of the RCTs. Still, it can be difficult to draw conclusions given the multiple ways this information was collected and reported, including as a binary, categorical, or continuous variable. Most observational studies had participants with a high educational status, as defined by each study. Similarly, many studies did not report information on ethnicity or race for their participants, but of the studies that did, the majority studied predominantly white individuals or individuals with European/Caucasian ethnicity. Most of the research was conducted in cohorts based in Europe and North America. Of note, Dehghan et al. (146) surveyed from 21 countries on five continents, presenting a more geographically diverse group of participants. Similarly, the racial and ethnic diversity of the participant cohorts in the RCTs is minimal. Most studies comprised of predominantly Caucasian individuals and were conducted in either primarily Canada or European countries. More diversity in participant cohorts in terms of age, education, race, and ethnicity is warranted to determine the effects of dairy fat from different products on cardiometabolic health in all people, not just a portion of the population.

Over the past decade, research and public efforts have accelerated rapidly to better understand the impact of different foods and dietary patterns beyond health outcomes, and to now further examine how diets can contribute to achieving food system transformation. A guiding framework for characterizing a sustainable food system comprises four interrelated domains: health, environment, economics, and society (178). Of note, this review sought to evaluate the role of dairy fat and its matrix on human health, and is therefore centralized within one out of the four pillars (i.e., health) of a sustainable food system; yet, all foods, including dairy foods, have different and complementary contributions to the four domains of sustainable food systems that deserve more research attention. For example, dairy farming contributes to greenhouse emissions through resources and energy to create food and ruminant livestock emission of methane gases; based on a 2008 life cycle assessment, dairy’s greenhouse gas footprint is an estimated 2% of the U.S. total (179). At the same time, dairy foods contribute essential nutrients to meet recommendations by the Food and Agriculture Organization of the United Nations, particularly for vulnerable populations including pregnant and lactating people, children and adolescence, and aging adults (180). Overall, much more research is warranted to better understand how dietary patterns that include plant and animal source foods, such as dairy foods, contribute to nutritionally adequate diets that promote human health as well as overall planetary health (181).

## 5 Conclusion

Our goal was to characterize the dairy-fat matrix across milk, yogurt, cheese, and butter, then examine their individual effects on cardiometabolic health to provide a critical assessment of the current body of science on the cardiometabolic health effects of dairy fat, evaluating gaps and implications in dietary guidance. Dairy fat is comprised of three increasingly complex levels of organization, starting with the FA and building to the MFG, which gives rise to unique physical and chemical properties. Further, these properties are altered by the processing methods used to create the dairy products commonly available. We aimed to evaluate if these alterations would, by way of influences on the bioavailability, digestion, absorption, and/or metabolism of dairy fat, result in unique health effects of the individual dairy-fat matrices on cardiometabolic health. Many authors have addressed this complex question using a variety of methodologies, and though multifaceted approaches are appropriate for a multifaceted question, the lack of comparable methods creates barriers in drawing clear conclusions on whether and to what degree the dairy-fat matrix of different dairy products may impact cardiometabolic health. Overall, we found that the evidence largely suggests no effect of consuming higher-fat varieties of dairy products on cardiometabolic health, with minor differences between individual dairy products, when stratified by both dairy product and fat content. More broadly, the current body of evidence suggests that regular fat dairy products may be a part of overall healthy eating patterns, but the variability in results suggest ample opportunity to bolster the current evidence base. Our recommendations for future research include replicable study designs that incorporate more variety in dairy product types and greater age, educational, socioeconomic, and racial/ethnic diversity within participant cohorts to create a cohesive literature base for dietary recommendations. Moving forward, conducting research with an understanding of the physical and chemical influences imparted by the individual dairy-fat matrices in dairy products will allow for more robust and comprehensive knowledge on the effect of the consumption of each of these unique, regular-fat dairy products on cardiometabolic health and their role in a healthy diet.

## Author contributions

VT: Data curation, Formal analysis, Investigation, Methodology, Visualization, Writing – original draft. AU: Conceptualization, Methodology, Writing – review & editing. JK: Funding acquisition, Conceptualization, Methodology, Project administration, Supervision, Writing – review & editing.
