# Supplementary material for: Full-fat dairy products and cardiometabolic health outcomes: Does the dairy-fat matrix matter?
Source: Front Nutr. 2024 Jul 29;11:1386257. doi: 10.3389/fnut.2024.1386257 (PMC11317386; doi:10.3389/fnut.2024.1386257)
Supplement: Supplementary file 1 [file Table_1.pdf]

Supplementary Material

Supplemental Table 1. Composition (g/100 g fatty acid methyl ester) of key fatty acids in cow's milk, yogurt, cheese, and butter reported in recent publications.

Fatty acid	Names	Milk	Yogurt	Cheese	Butter
SFAs		56.77-68.13 (1–8)	57.68-73.64 (9–12)	53.25-67.32 (4,6,13–17)	48.66 (18)
4:0	Butyric acid<br>Butanoic acid	1.36-4.28 (1,3,6–8,19–23)	0.67-4.58 (9,10,12)	2.47-3.62 (6,13,14,16,17,24)	1.34-4.00 (25–29)
6:0	Caproic acid<br>Hexanoic acid	1.56-2.26 (1,3,6–8,19–23)	1.90-2.85 (9,10,12)	1.71-2.06 (6,13,14,16,17,24)	1.85-2.60 (25–29)
8:0	Caprylic acid<br>Octanoic acid	0.97-1.35 (1,3,6–8,19–23)	0.77-1.69 (9,10,12)	1.03-1.34 (6,13,14,16,17,24)	1.02-2.04 (25–29)
10:0	Capric acid<br>Decanoic acid	1.96-4.07 (1,3,6–8,19–23)	1.92-3.91 (9,10,12)	2.06-3.13 (6,13,14,16,17,24)	2.29-4.77 (25–29)
12:0	Lauric acid<br>Dodecanoic acid	2.30-3.88 (1,3,6–8,19–23)	2.51-4.37 (9,10,12)	2.42-3.65 (6,13,14,16,17,24)	2.62-5.83 (25–29)
13:0 *iso*	11-Methyldodecanoic acid<br>*iso*-Tridecanoic acid	0.03-0.07 (1,3,6,23)	No data available	0.07-0.10 (6,16,24)	No data available
14:0	Myristic acid<br>Tetradecanoic acid	8.77-11.97 (1,3,6–8,19–23)	9.26-13.18 (9,10,12)	8.36-11.71 (6,13,14,16,24)	4.02-11.86 (25–29)
15:0	Pentadecanoic acid<br>Pentadecylic acid	0.95-1.50 (1,3,6–8,19–23)	0.99-1.47 (9,10,12)	0.92-1.34 (6,13,14,16,17,24)	0.90-1.71 (25–29)
15:0 *iso*	13-Methyltetradecanoic acid<br>*iso*-Pentadecanoic acid	0.13-0.36 (3,6,22,23,30)	0.15 (30)	0.16-0.43 (6,16,24,30)	0.01 (30)
15:0 *anteiso*	13-Methylpentadecanoic acid<br>*anteiso*-Pentadecanoic acid	0.43-0.64 (1,3,6,20,22,23,30)	0.63 (30)	0.54-0.60 (6,16,24,30)	0.63 (30)

16:0	Palmitic acid<br>Hexadecanoic acid	22.81-32.33<br>(1,3,31,6–8,19–23)	26.37-31.70<br>(9,10,12)	23.47-30.81<br>(6,13,14,16,17,24)	20.92-32.59<br>(25–29)
17:0	Margaric acid<br>Heptadecanoic acid	0.49-0.74<br>(1,3,6–8,19–23)	0.57-0.87<br>(9,10,12)	0.58-0.86<br>(6,13,16,17,24)	0.45-0.83<br>(13,25,27,28)
17:0 *iso*	15-Methylhexadecanoic acid<br>*iso*-Heptadecanoic acid	0.26-0.41<br>(1,3,6,20,22,23,30)	0.21<br>(30)	0.22-0.43<br>(6,16,24,30)	0.31<br>(30)
17:0 *anteiso*	14-Methylhexadecanoic acid<br>*anteiso*-Heptadecanoic acid	0.35-0.64<br>(3,6,20,22,23,30)	0.58<br>(30)	0.21-0.48<br>(6,16,24,30)	0.38<br>(30)
18:0	Stearic acid<br>Octadecanoic acid	10.02-11.27<br>(1,3,31,6–8,19–23)	7.94-11.82<br>(9,10,12)	6.83-11.17<br>(6,13,14,16,17,24)	6.71-13.25<br>(25–29)
MUFAs		24.14-35.11<br>(1–8,31)	22.69-31.48<br>(9–12)	19.05-33.83<br>(4,6,13–17)	15.03-25.48<br>(18,27)
14:1 *c*9	Myristoleic acid<br>9-*cis*-Tetracenoic acid	0.82-1.36<br>(1,3,6,7,19–23)	1.04-1.48<br>(10,12)	0.26-0.96<br>(6,13,17,32)	0.36-1.66<br>(13,27,32)
16:1 *t*9	*trans*-Palmitoleic acid<br>9-*trans*-Hexadecenoic acid	0.01-0.40<br>(1,3,6,7,22,23,33)	0.03-0.44<br>(9,33)	0.04-0.25<br>(32,33)	0.02-0.07<br>(32,33)
16:1 *c*9	Palmitoleic acid<br>9-*cis*-Hexadecenoic acid	1.22-3.70<br>(3,6–8,19–23)	1.87-2.30<br>(9,10,12)	1.33-1.54<br>(6,13,17)	1.11-2.59<br>(13,25,27)
18:1 *t*11	Vaccenic acid<br>11-*cis*-Octadecenoic acid	0.77-4.54<br>(1,3,6,19,20,22,23,31,33)	1.37-1.60<br>(9,12,33)	1.41-4.31<br>(13,17,32,33)	0.80-1.32<br>(32,33)
18:1 *c*9	Oleic acid<br>9-*cis*-Octadecenoic acid	15.94-24.74<br>(1,6,19–23,31)	14.01-26.89<br>(9,10,12)	14.17-23.67<br>(6,13,14,16,17,24)	12.62-20.69<br>(13,27)
PUFAs		3.43-6.18<br>(1–8,31)	2.45-3.23<br>(9–12)	3.05-5.82<br>(4,6,13–17)	2.56-5.30<br>(18,27)
18:2 *c*9,*t*11	Rumenic acid<br>9-*cis*,11-*trans*-Octadecadienoic acid	0.41-1.74<br>(1,3,6,19–23,31)	0.54-0.82<br>(9,10,12)	0.65-1.47<br>(6,13,16,17,24)	0.71-1.21<br>(13,26)
18:2 *c*9,*c*12	Linoleic acid<br>9-*cis*,12-*cis*-Octadecadienoic acid	0.86-3.16<br>(1,3,31,6–8,19–23)	0.25-2.23<br>(9,10,12)	0.88-2.44<br>(6,13,14,16,17,24)	0.76-4.66<br>(13,25,27)
18:3 *c*9,*c*12,*c*15	α-Linolenic acid	0.32-1.00	0.39-0.59	0.44-0.91	0.52-0.64

	9-cis,12-*cis*,15-cis-Octadecatrienoic acid	(1,3,31,6–8,19–23)	(9,10,12)	(6,13,16,17,24)	(13,25,27)
20:4 *c*5,*c*8,*c*11,*c*14	Arachidonic acid 5-*cis*,8-*cis*,11-*cis*,14-*cis*-Eicosatetraenoic acid	0.01-0.20 (1,3,6–8,19,20,23)	0.20-0.32 (9,10)	0.04-0.16 (6,13)	0.05-0.10 (13,25)
20:5 *c*5,*c*8,*c*11,*c*14,*c*17	Timnodonic acid 5-*cis*,8-*cis*,11-*cis*,14-*cis,*17-*cis*-Eicosapentaenoic acid	0.01-0.09 (1,3,6,7,19,21,23,31)	0.14-0.23 (9,10)	0.09 (6)	0.05 (25)
22:6 *c*4,*c*7,*c*10,*c*13,*c*16,*c*19	Cervonic acid 4-*cis*,7-*cis*,10-*cis*,13-*cis,*16-*cis*, 19-*cis*-Docosahexaenoic acid	Not detected-0.03 (1,21,23,31)	0.09-0.19 (9,10)	No data available	0.00 (25)

References

1. Stergiadis S, Berlitz CB, Hunt B, Garg S, Givens DI, Kliem KE. An update to the fatty acid profiles of bovine retail milk in the United Kingdom: Implications for nutrition in different age and gender groups. *Food Chem* (2019) 276:218–230. doi: 10.1016/j.foodchem.2018.09.165

2. Hernández-Ortega M, Martínez-Fernández A, Soldado A, González A, Arriaga-Jordán CM, Argamentería A, De La Roza-Delgado B, Vicente F. Effect of total mixed ration composition and daily grazing pattern on milk production, composition and fatty acids profile of dairy cows. *J Dairy Res* (2014) 81:471–478. doi: 10.1017/s0022029914000399

3. Henno M, Ariko T, Kaart T, Kuusik S, Ling K, Kass M, Jaakson H, Leming R, Givens DI, Sterna V, et al. The fatty acid composition of Estonian and Latvian retail milk; implications for human nutrition compared with a designer milk. *J Dairy Res* (2018) 85:247–250. doi: 10.1017/s0022029918000183

4. Corazzin M, Romanzin A, Sepulcri A, Pinosa M, Piasentier E, Bovolenta S. Fatty acid profiles of cow's milk and cheese as affected by mountain pasture type and concentrate supplementation. *Animals* (2019) 9:68. doi: 10.3390/ani9020068

5. Cozma A, Martin B, Cirié C, Verdier-Metz I, Agabriel J, Ferlay A. Influence of the calf presence during milking on dairy performance, milk fatty acid composition, lipolysis and cheese composition in Salers cows during winter and grazing seasons. *J Anim Physiol Anim Nutr (Berl)* (2017) 101:949–963. doi: 10.1111/jpn.12530

6. Bergamaschi M, Bittante G. Detailed fatty acid profile of milk, cheese, ricotta and by products, from cows grazing summer highland pastures. *J Dairy Res* (2017) 84:329–338. doi: 10.1017/s0022029917000450

7. Benbrook CM, Butler G, Latif MA, Leifert C, Davis DR. Organic production enhances milk nutritional quality by shifting fatty acid composition: A United States–wide, 18-month study. *PLoS One* (2013) 8:e82429. doi: 10.1371/journal.pone.0082429

8. Liu N, Pustjens AM, Erasmus SW, Yang Y, Hettinga K, van Ruth SM. Dairy farming system markers: The correlation of forage and milk fatty acid profiles from organic, pasture and conventional systems in the Netherlands. *Food Chem* (2020) 314:126153. doi: 10.1016/j.foodchem.2019.126153

9. Caglayan O, Cakmak YS, Guler GO, Zengin G, Aktumsek A. Evaluation of fatty acid compositions of yogurts in Turkey. *Asian J Chem* (2014) 26:4871–4874. doi: 10.14233/ajchem.2014.16323

10. Serafeimidou A, Zlatanos S, Laskaridis K, Sagredos A. Chemical characteristics, fatty acid composition and conjugated linoleic acid (CLA) content of traditional Greek yogurts. *Food Chem* (2012) 134:1839–1846. doi: 10.1016/j.foodchem.2012.03.102

11. Serafeimidou A, Zlatanos S, Kritikos G, Tourianis A. Change of fatty acid profile, including conjugated linoleic acid (CLA) content, during refrigerated storage of yogurt made of cow and

sheep milk. *J Food Compos Anal* (2013) 31:24–30. doi: 10.1016/j.jfca.2013.02.011

12. Akbaridoust G, Plozza T, Trenerry VC, Wales WJ, Auldist MJ, Ajlouni S. Influence of pasture-based feeding systems on fatty acids, organic acids and volatile organic flavour compounds in yoghurt. *J Dairy Res* (2015) 82:279–286. doi: 10.1017/s0022029915000357

13. O'Callaghan TF, Mannion DT, Hennessy D, McAuliffe S, O'Sullivan MG, Leeuwendaal N, Beresford TP, Dillon P, Kilcawley KN, Sheehan JJ, et al. Effect of pasture versus indoor feeding systems on quality characteristics, nutritional composition, and sensory and volatile properties of full-fat Cheddar cheese. *J Dairy Sci* (2017) 100:6053–6073. doi: 10.3168/jds.2016-12508

14. Manuelian CL, Currò S, Penasa M, Cassandro M, De Marchi M. Characterization of major and trace minerals, fatty acid composition, and cholesterol content of Protected Designation of Origin cheeses. *J Dairy Sci* (2017) 100:3384–3395. doi: 10.3168/jds.2016-12059

15. Paszczyk B, Polak-Śliwińska M, Zielak-Steciwko AE. Chemical composition, fatty acid profile, and lipid quality indices in commercial ripening of cow cheeses from different seasons. *Animals* (2022) 12:198. doi: 10.3390/ani12020198

16. Paszczyk B, Łuczyńska J. The comparison of fatty acid composition and lipid quality indices in hard cow, sheep, and goat cheeses. *Foods* (2020) 9:1667. doi: 10.3390/foods9111667

17. Formaggioni P, Malacarne M, Franceschi P, Zucchelli V, Faccia M, Battelli G, Brasca M, Summer A. Characterisation of Formaggella della Valle di Scalve cheese produced from cows reared in valley floor stall or in mountain pasture: Fatty acids profile and sensory properties. *Foods* (2020) 9:383. doi: 10.3390/foods9040383

18. O'Callaghan TF, Faulkner H, McAuliffe S, O'Sullivan MG, Hennessy D, Dillon P, Kilcawley KN, Stanton C, Ross RP. Quality characteristics, chemical composition, and sensory properties of butter from cows on pasture versus indoor feeding systems. *J Dairy Sci* (2016) 99:9441–9460. doi: 10.3168/jds.2016-11271

19. O'Donnell AM, Spatny KP, Vicini JL, Bauman DE. Survey of the fatty acid composition of retail milk differing in label claims based on production management practices. *J Dairy Sci* (2010) 93:1918–1925. doi: 10.3168/jds.2009-2799

20. Tzamaloukas O, Neofytou MC, Simitzis PE, Miltiadou D. Effect of farming system (organic vs. conventional) and season on composition and fatty acid profile of bovine, caprine and ovine milk and retail halloumi cheese produced in Cyprus. *Foods* (2021) 10:1016. doi: 10.3390/foods10051016

21. Capuano E, Gravink R, Boerrigter-Eenling R, van Ruth SM. Fatty acid and triglycerides profiling of retail organic, conventional and pasture milk: Implications for health and authenticity. *Int Dairy J* (2015) 42:58–63. doi: 10.1016/j.idairyj.2014.11.002

22. Heck JML, van valenberg HJF, Dijkstra J, van Hooijdonk ACM. Seasonal variation in the Dutch bovine raw milk composition. *J Dairy Sci* (2009) 92:4745–4755. doi: 10.3168/jds.2009-2146

23. Schwendel BH, Morel PCH, Wester TJ, Tavendale MH, Deadman C, Fong B, Shadbolt NM,

Thatcher A, Otter DE. Fatty acid profile differs between organic and conventionally produced cow milk independent of season or milking time. *J Dairy Sci* (2015) 98:1411–1425. doi: 10.3168/jds.2014-8322

24. Paszczyk B, Łuczyńska J, Polak-Śliwińska M. The effect of storage on the yogurt fatty acid profile. *Mljekarstvo* (2020) 70:59–70. doi: 10.15567/mljekarstvo.2020.0106

25. Pustjens AM, Boerrigter-Eenling R, Koot AH, Rozijn M, Ruth SM van. Characterization of retail conventional, organic, and grass full-fat butters by their fat contents, free fatty acid contents, and triglyceride and fatty acid profiling. *Foods* (2017) 6:26. doi: 10.3390/foods6040026

26. Pujolras MP, Ayvaz H, Shotts ML, Pittman RA, Herringshaw S, Rodriguez-Saona LE. Portable infrared spectrometer to characterize and differentiate between organic and conventional bovine butter. *J Am Oil Chem Soc* (2015) 92:175–184. doi: 10.1007/s11746-015-2591-x

27. Pădureţ S. The effect of fat content and fatty acids composition on color and textural properties of butter. *Molecules* (2021) 26:4565. doi: 10.3390/molecules26154565

28. Staniewski B, Ogrodowska D, Staniewska K, Kowalik J. The effect of triacylglycerol and fatty acid composition on the rheological properties of butter. *Int Dairy J* (2021) 114:104913. doi: 10.1016/j.idairyj.2020.104913

29. O'Callaghan TF, Faulkner H, McAuliffe S, O'Sullivan MG, Hennessy D, Dillon P, Kilcawley KN, Stanton C, Ross RP. Corrigendum to "Quality characteristics, chemical composition, and sensory properties of butter from cows on pasture versus indoor feeding systems" (J. Dairy Sci. 99:9441–9460). *J Dairy Sci* (2018) 101:8616. doi: 10.3168/jds.2018-101-9-8616

30. Ran-Ressler RR, Bae S, Lawrence P, Wang DH, Brenna JT. Branched-chain fatty acid content of foods and estimated intake in the USA. *Br J Nutr* (2014) 112:565–572. doi: 10.1017/s0007114514001081

31. Unger AL, Bourne DE, Walsh H, Kraft J. Fatty acid content of retail cow's milk in the northeastern United States-What's in it for the consumer? *J Agric Food Chem* (2020) 68:4268–4276. doi: 10.1021/acs.jafc.9b07390

32. Hauff S, Vetter W. Quantification of branched chain fatty acids in polar and neutral lipids of cheese and fish samples. *J Agric Food Chem* (2010) 58:707–712. doi: 10.1021/jf9034805

33. Guillocheau E, Penhoat C, Drouin G, Godet A, Catheline D, Legrand P, Rioux V. Current intakes of trans-palmitoleic (trans-C16:1 n-7) and trans-vaccenic (trans-C18:1 n-7) acids in France are exclusively ensured by ruminant milk and ruminant meat: A market basket investigation. *Food Chem X* (2020) 5:100081. doi: 10.1016/j.fochx.2020.100081
