# Supplementary material for: Full-fat dairy products and cardiometabolic health outcomes: Does the dairy-fat matrix matter?
Source: Front Nutr. 2024 Jul 29;11:1386257. doi: 10.3389/fnut.2024.1386257 (PMC11317386; doi:10.3389/fnut.2024.1386257)
Supplement: Supplementary file 2 [file Table_2.pdf]

Supplementary Material

Supplemental Table 2. Summary of observational studies (n = 46) evaluating the effects of dairy fat from milk, yogurt, cheese, and/or butter on cardiometabolic disease outcomes.

Reference	Dairy Food	Population	Study design	Methods for Dietary Survey	Analysis type
Slurink et al. (1)	Milk<br>-High-fat (>2%)<br>-Low-fat (≤2%)<br>Cheese<br>-High-fat (>20%)<br>-Low-fat (≤20%)	Australian adults<br>n = 4891<br>>25 years	Prospective; follow-up of 12 years	FFQ[a] over the previous year assessed at baseline, at 5 years, and at 12 years	Comparison between intake levels of high-fat food[b]
Van Parys et al. (2)	Butter	Norwegian adults with stable angina pectoris<br>n = 1929<br>>18 years	Prospective; median follow-up of 5.2 and 7.8 years for stroke and acute myocardial infarction, respectively	FFQ over the previous year assessed at baseline	Comparison between intake levels of high-fat food
Slurink et al. (3)	Milk<br>-High-fat (>2%)<br>Cheese<br>-High-fat (>20%)	Dutch adults<br>n = 2,262<br>40-75 years	Prospective cohort; mean follow-up of 6.4 years	FFQ over the previous year assessed at baseline and at 7 years	Comparison between intake levels of high-fat food
McGovern et al. (4)	Milk<br>-Higher-fat [Whole (3.25%) and 2%]	U.S. American children<br>n = 796	Prospective cohort; mean	FFQ over the previous month assessed at early childhood visit (mean age 3.2 years)	Comparison between intake of low- and high-fat foods[c]

	-Lower-fat [1% and skim)	Followed from birth	follow-up 13.1 years		
Wang et al. (5)	Milk<br>-Whole (3-4%)<br>-Low-fat (2% and ≤1%)	U.S. American adults<br>n = 29,283<br>≥20 years	Prospective cohort; median follow-up of 8.3 years	Milk intake assessed over the past month at baseline	Comparison between intake of low- and high-fat foods
Ibsen et al. (6)	Milk<br>-Whole (3.5%)<br>-Semi-skim (1.5%)<br>-Skim (0.1%)<br>Yogurt<br>-Whole (3.5%)<br>-Low-fat (<1.5%)	Danish adults<br>n = 39,393<br>56-72 years	Prospective; follow-up of 10 years	FFQ assessed over the previous year and 5 years later	Substitution analysis[d]
Cruijsen et al. (7)	Milk<br>-High-fat (≥3.5%)<br>Butter	Dutch adults with history of myocardial infarction within previous 10 years<br>n = 4,365<br>60-80 years	Prospective; follow-up of 12-16 years	FFQ over the previous month assessed at baseline	Comparison between intake levels of high-fat food
Shi et al. (8)	Cheese<br>-Full-fat (not defined)<br>Butter	U.S. American postmenopausal women<br>n = 35352<br>50-79 years	Cross-sectional	FFQ over the previous three months assessed at baseline	Comparison between intake levels of high-fat food
Machlik et al. (9)	Yogurt<br>-Regular-fat (not defined)<br>Cheese<br>-Regular-fat (not defined)	Norwegian adults<br>n = 10844<br>≥40 years	Cross-sectional	FFQ over the previous year	Comparison between intake levels of high-fat food

Wilkinson et al. (10)	Milk<br>-Full-fat (not defined)<br>-2% and 1%<br>-Non-fat/skim	U.S. American adults<br>n = 13,544<br>20-79 years	Cross-sectional	Milk intake assessed over the past month from NHANES 2011-2016 interviews	Comparison between intake of low- and high-fat foods
Zhang et al. (11)	Butter	U.S. American adults<br>n = 521,120<br>50-71 years	Prospective; mean follow-up of 16 years	FFQ over the previous year assessed at baseline	Comparison between intake levels of high-fat food
Kvist et al. (12)	Milk<br>-High-fat (3.5%)<br>-Low-fat [skim (0.3%) and semi-skim (1.5%)]<br>Yogurt<br>-High-fat (3.5%)<br>-Low-fat (1.5%)	Danish adults without myocardial infarction diagnosis<br>n = 59,403<br>50-64 years	Prospective; median follow-up of 15.9 years	FFQ over the previous year assessed at baseline	Substitution analysis
White et al. (13)	Milk<br>-Whole (4%)<br>-2% and 1%<br>-Fat free	U.S. American children<br>n = 8,367<br>2-6 years<br>U.S. American children, adolescents, and young adults<br>n = 26,750<br>2-20 years	Cross-sectional	Milk intake assessed from repeated cross sections of NHANES; current intake assessed over the past month	Comparison between intake of low- and high-fat foods
Trichia et al. (14)	Yogurt<br>-Full-fat (≥3.9%)<br>Cheese<br>-High-fat (≥3.9%)<br>Butter	British adults<br>n = 15612<br>40-78 years	Prospective cohort; mean follow-up of 3.7 years	FFQ over the previous year assessed at baseline and follow-up	Comparison between intake levels of high-fat food

Lahoz-García et al. (15)	Milk<br>-Whole (not defined)	Spanish children<br>n = 1088<br>8-11 years	Cross-sectional	FFQ over the previous week	Comparison between intake levels of high-fat food
Ding et al. (16)	Milk<br>-Whole (not defined)	U.S. American adults<br>n = 217,755<br>25-75 years	Prospective cohort; follow-up of 29-32 years	FFQ over the previous year assessed at baseline and every 4 years	Comparison between intake levels of high-fat food
Drouin-Chartier et al. (17)	Milk<br>-Whole (not defined)<br>-Reduced-fat (skim, 1-2%)	U.S. American adults<br>n = 192,352<br>25-75 years	Prospective cohort; follow-up of 29-32 years	FFQ over the previous year assessed at baseline and every 4 years	Comparison between intake levels of high-fat food; Substitution analysis
Wong et al. (18)	Milk<br>-Whole (3.25%)<br>-2% and 1%<br>-Skim	Canadian children<br>n = 2,890<br>2-8 years	Cross-sectional	Parent-reported daily milk consumption	Comparison between intake of low- and high-fat foods
Kummer et al. (19)	Milk<br>-Full-fat (not defined)	U.S. American Indian adults<br>n = 1,623<br>14-86 years	Prospective cohort; mean follow-up of 11 years	FFQ over the previous year assessed at baseline	Comparison between intake levels of high-fat food
Laursen et al. (20)	Milk<br>-High-fat (≥3%)<br>-Low-fat (<2%)<br>Yogurt<br>-High-fat (≥3%)<br>-Low-fat (<2%)<br>Butter	Dutch adults<br>n = 36,886<br>21-70 years	Prospective; median follow-up of 15.2 years	FFQ over the previous year	Substitution analysis
Johansson et al. (21)	Milk<br>-High-fat (3.0%)<br>-Medium-fat (1.5%)<br>-Low-fat (0.5%)	Swedish adults<br>n = 98,572<br>30-60 years	Prospective cohort; mean follow-up of 14.2 years	FFQ over the previous year assessed annually	Comparison between intake levels of high-fat food

	Cheese<br>-High-fat (≥28%)<br>Butter				
Koskinen et al. (22)	Milk<br>-High-fat (≥3.5%)<br>Cheese<br>-High-fat (≥3.5%)<br>Butter	Finnish men<br>n = 1,981<br>42-60 years	Prospective; mean follow-up of 20.1 years	Four-day food record assessed at baseline	Comparison between intake levels of high-fat food
Johansson et al. (23)	Milk<br>-High-fat (3.0%)<br>-Medium-fat (1.5%)<br>-Low-fat (0.5%)<br>Cheese<br>-≥28% fat<br>Butter	Swedish adults<br>n = 90,512<br>30-60 years	Prospective cohort; follow-up of 8-12 years; cross-sectional analysis also performed	FFQ over the previous year assessed annually	Comparison between intake levels of high-fat food; Comparison between intake of low- and high-fat foods
Dehghan et al. (24)	Butter	Adults from 21 countries<br>n = 136,384<br>35-70 years	Prospective; follow-up of 9.1 years	FFQ over the past year assessed at baseline	Comparison between intake levels of high-fat food
Sun et al. (25)	Milk<br>-Whole (not defined)<br>-Low/reduced-fat (not defined)<br>-Skim (not defined)	Australian adults<br>n = 2,071<br>26-36 years	Cross-sectional	FFQ and food habits questionnaire over the previous year	Comparison between intake of low- and high-fat foods
Brouwer-Brolsma et al. (26)	Milk<br>-Full-fat (3.5%)<br>Yogurt<br>-Full-fat (2.9%)<br>Cheese<br>-Full-fat (≥24%)	Dutch adults<br>n = 112,086<br>≥18 years	Cross-sectional	FFQ over the previous month	Comparison between intake levels of high-fat food

Laursen et al. (27)	Milk<br>-Full-fat (3.5%)<br>-Low-fat [skim (0.3%) and semi-skim (1.5%)]	Danish adults without previous stroke<br>n = 55,211<br>50-64 years	Prospective; mean follow-up of 13.4 years	FFQ over the previous year assessed at baseline	Substitution analysis
Um et al. (28)	Milk<br>-Whole (not defined)	U.S. American adults<br>n = 21,427<br>≥45 years	Prospective; follow-up of 11 years	FFQ over the previous year assessed at baseline	Comparison between intake levels of high-fat food
Hruby et al. (29)	Milk<br>-Whole (not defined)	U.S. American adults<br>n = 2,809<br>44-64 years	Prospective; mean follow-up of 12 years	FFQ over the previous year assessed at baseline and at three additional timepoints	Comparison between intake levels of high-fat food
Guasch-Ferré et al. (30)	Milk<br>-Whole (not defined)<br>Yogurt<br>-Whole (not defined)<br>Butter	Spanish adults at high risk for cardiovascular diseases without type 2 diabetes<br>n = 3,349<br>55-80 years	Prospective; mean follow-up of 4.3 years	FFQ assessed at baseline and yearly	Comparison between intake levels of high-fat food
Karatzi et al. (31)	Cheese<br>-High-fat/regular (not defined)	Greek adults at risk of cardiovascular diseases<br>n = 181<br>≥18 years	Cross-sectional	24-hour recalls over one weekday and one weekend day	Comparison between intake levels of high-fat food
Brouwer-Brolsma et al. (32)	Cheese<br>-Full-fat (≥50%)<br>Butter	Dutch adults<br>n = 2,974<br>≥55 years	Prospective cohort; mean follow-up of 9.5 years	FFQ assessed at baseline	Comparison between intake levels of high-fat food

Santiago et al. (33)	Yogurt<br>-Whole (not defined)	Spanish adults at high risk for cardiovascular diseases<br>n = 4,545<br>55-80 years	Prospective cohort; median follow-up of 4.9 years	FFQ over the previous year assessed at baseline and yearly	Comparison between intake levels of high-fat food
Díaz-López et al. (34)	Milk<br>-Whole (not defined)<br>Yogurt<br>-Whole (not defined)	Spanish adults at high risk for cardiovascular diseases without type 2 diabetes<br>n = 3,454<br>55-80 years	Prospective cohort; median follow-up of 4.1 years	FFQ assessed at baseline and yearly	Comparison between intake levels of high-fat food
Drehmer et al. (35)	Butter	Brazilian adults<br>n = 9,835<br>35-74 years	Cross-sectional	FFQ over the previous year	Comparison between intake levels of high-fat food
Hosseinpour-Niazi et al. (36)	Butter	Iranian adults<br>n = 1,582<br>19-84 years	Prospective cohort; follow-up of 3 years	FFQ over the previous year assessed at baseline	Comparison between intake levels of high-fat food
Drehmer et al. (37)	Butter	Brazilian adults<br>n = 10,010<br>35-74 years	Cross-sectional	FFQ over the previous year assessed	Comparison between intake levels of high-fat food
Ericson et al. (38)	Milk<br>-High-fat (≥2.5% fat)	Swedish adults<br>n = 26,930<br>45-74 years	Prospective cohort; mean follow-up of 14 years	Seven-day food record, FFQ over previous year, and interview assessed at baseline	Comparison between intake levels of high-fat food
Buijsse et al. (39)	Butter	European adults<br>n = 25,307<br>≥20 years	Prospective cohort; median follow-up of 12.3 years	Country specific FFQ over the previous year assessed at baseline	Comparison between intake levels of high-fat food

Sayón-Orea et al. (40)	Yogurt<br>-Whole (not defined)	Spanish adults<br>n = 8,063<br>20-90 years	Prospective cohort; follow-up of 6 years	FFQ over the previous year assessed at baseline	Comparison between intake levels of high-fat food
Crichton et al. (41)	Milk<br>-Whole (not defined)<br>Yogurt<br>-Regular-fat/Whole (not defined)<br>Cheese<br>-Regular-fat/Whole (not defined)<br>Butter	Luxembourger adults<br>n = 1,352<br>18-69 years	Cross-sectional	FFQ over the previous 3 months	Comparison between intake levels of high-fat food
Crichton et al. (42)	Milk<br>-Whole (not defined)<br>Yogurt<br>-Regular-fat/Whole (not defined)<br>Cheese<br>-Regular-fat/Whole	Luxembourger adults<br>n = 1,352<br>18-69 years	Cross-sectional	FFQ over the previous 3 months	Comparison between intake levels of high-fat food
Avalos et al. (43)	Milk<br>-Whole (not defined)<br>Butter	U.S. American adults<br>n = 1,759<br>≥50 years	Prospective cohort; follow-up of 20 years	FFQ over the previous year assessed at baseline	Comparison between intake levels of high-fat food
Patterson et al. (44)	Milk<br>-Full-fat (≥3%)<br>-Low-fat [Semi-skin (≤1.5%) and Skim (0.5%)]<br>Cheese<br>-Full-fat (>17%)	Swedish women<br>n = 33,636<br>48-83 years	Prospective cohort; mean follow-up of 11.6 years	FFQ over the previous year	Comparison between intake levels of high-fat food; Substitution analysis

	-Low-fat (10-17%)				
Holmberg et al. (45)	Milk<br>-Full-fat/high-fat (3.0% fat)<br>-Low-fat (≤1.5% fat)	Swedish men<br>n = 1,782<br>40-60 years	Prospective cohort; follow-up of 12 years	FFQ over the previous week assessed at baseline	Comparison between intake of low- and high-fat foods
Scharf et al. (46)	Milk<br>-High-fat (Whole/2%)<br>-Low-fat (1%/skim)	U.S. American children<br>n = 7,450 at 2-year examination; 8,300 at 4-year examination<br>0-5 years	Prospective cohort; follow-up of 5 years	Parent-reported milk consumption assessed at years 2 and 4	Comparison between intake levels of high-fat food; Comparison between intake of low- and high-fat foods

[a]FFQ = food frequency questionnaire. [b]Example: comparing the effect of one versus three daily servings of regular-fat milk on a given cardiometabolic health outcome. [c]Example: comparing the effect of non-fat versus regular-fat milk on a given cardiometabolic health outcome. [d]Example: modeling the estimated effect of substituting non-fat milk with regular-fat milk, on a given cardiometabolic health outcome.

References

1. Slurink I AL, Chen L, Magliano DJ, Kupper N, Smeets T, Soedamah-Muthu SS. Dairy product consumption and incident prediabetes in the Australian Diabetes, Obesity, and Lifestyle Study with 12 years of follow-up. *J Nutr* (2023) 153:1742–1752. doi: 10.1016/j.tjnut.2023.03.032

2. Van Parys A, Sæle J, Puaschitz NG, Anfinsen ÅM, Karlsson T, Olsen T, Haugsgjerd TR, Vinknes KJ, Holven KB, Dierkes J, et al. The association between dairy intake and risk of cardiovascular disease and mortality in patients with stable angina pectoris. *Eur J Prev Cardiol* (2023) 30:219–229. doi: 10.1093/eurjpc/zwac217

3. Slurink IAL, den Braver NR, Rutters F, Kupper N, Smeets T, Elders PJM, Beulens JWJ, Soedamah-Muthu SS. Dairy product consumption and incident prediabetes in Dutch middle-aged adults: the Hoorn Studies prospective cohort. *Eur J Nutr* (2022) 61:183. doi: 10.1007/s00394-021-02626-9

4. McGovern C, Rifas-Shiman SL, Switkowski KM, Woo Baidal JA, Lightdale JR, Hivert MF, Oken E, Aris IM. Association of cow's milk intake in early childhood with adiposity and cardiometabolic risk in early adolescence. *Am J Clin Nutr* (2022) 116:561–571. doi: 10.1093/ajcn/nqac103

5. Wang S, Liu Y, Cai H, Li Y, Zhang X, Liu J, Sun R, Fang S, Yu B. Decreased risk of all-cause and heart-specific mortality is associated with low-fat or skimmed milk consumption compared with whole milk intake: A cohort study. *Clin Nutr* (2021) 40:5568–5575. doi: 10.1016/j.clnu.2021.09.012

6. Ibsen DB, Overvad K, Laursen ASD, Halkjær J, Tjønneland A, Kilpeläinen TO, Parner ET, Jakobsen MU. Changes in intake of dairy product subgroups and risk of type 2 diabetes: modelling specified food substitutions in the Danish Diet, Cancer and Health cohort. *Eur J Nutr* (2021) 60:3449–3459. doi: 10.1007/s00394-021-02524-0

7. Cruijsen E, Jacobo Cejudo MG, Küpers LK, Busstra MC, Geleijnse JM. Dairy consumption and mortality after myocardial infarction: A prospective analysis in the Alpha Omega Cohort. *Am J Clin Nutr* (2021) 114:59–69. doi: 10.1093/ajcn/nqab026

8. Shi N, Olivo-Marston S, Jin Q, Aroke D, Joseph JJ, Clinton SK, Manson JAE, Rexrode KM, Mossavar-Rahmani Y, Fels Tinker L, et al. Associations of dairy intake with circulating biomarkers of inflammation, insulin response and dyslipidemia among postmenopausal women. *J Acad Nutr Diet* (2021) 121:1984–2002. doi: 10.1016/j.jand.2021.02.029

9. Machlik ML, Hopstock LA, Wilsgaard T, Hansson P. Associations between intake of fermented dairy products and blood lipid concentrations are affected by fat content and dairy matrix – The Tromsø Study: Tromsø7. *Front Nutr* (2021) 8:773468. doi: 10.3389/fnut.2021.773468/bibtex

10. Wilkinson K, Tucker L, Davidson L, Bailey B. Milk-fat intake and differences in abdominal adiposity and BMI: Evidence based on 13,544 randomly-selected adults. *Nutrients* (2021)

13:1832. doi: 10.3390/nu13061832

11. Zhang Y, Zhuang P, Wu F, He W, Mao L, Jia W, Zhang Y, Chen X, Jiao J. Cooking oil/fat consumption and deaths from cardiometabolic diseases and other causes: Prospective analysis of 521,120 individuals. *BMC Med* (2021) 19:92. doi: 10.1186/s12916-021-01961-2

12. Kvist K, Laursen ASD, Overvad K, Jakobsen MU. Substitution of milk with whole-fat yogurt products or cheese is associated with a lower risk of myocardial infarction: The Danish Diet, Cancer and Health cohort. *J Nutr* (2020) 150:1252–1258. doi: 10.1093/jn/nxz337

13. White MJ, Armstrong SC, Kay MC, Perrin EM, Skinner A. Associations between milk fat content and obesity, 1999 to 2016. *Pediatr Obes* (2020) 15:e12612. doi: 10.1111/ijpo.12612

14. Trichia E, Luben R, Khaw KT, Wareham NJ, Imamura F, Forouhi NG. The associations of longitudinal changes in consumption of total and types of dairy products and markers of metabolic risk and adiposity: Findings from the European Investigation into Cancer and Nutrition (EPIC)–Norfolk study, United Kingdom. *Am J Clin Nutr* (2020) 111:1018–1026. doi: 10.1093/ajcn/nqz335

15. Lahoz-García N, Milla-Tobarra M, García-Hermoso A, Hernández-Luengo M, Pozuelo-Carrascosa DP, Martínez-Vizcaíno V. Associations between dairy intake, body composition, and cardiometabolic risk factors in Spanish schoolchildren: The Cuenca Study. *Nutrients* (2019) 11:2940. doi: 10.3390/nu11122940

16. Ding M, Li J, Qi L, Ellervik C, Zhang X, Manson JE, Stampfer M, Chavarro JE, Rexrode KM, Kraft P, et al. Associations of dairy intake with risk of mortality in women and men: Three prospective cohort studies. *BMJ* (2019) 367: doi: 10.1136/bmj.l6204

17. Drouin-Chartier JP, Li Y, Ardisson Korat AV, Ding M, Lamarche B, Manson JE, Rimm EB, Willett WC, Hu FB. Changes in dairy product consumption and risk of type 2 diabetes: Results from 3 large prospective cohorts of US men and women. *Am J Clin Nutr* (2019) 110:1201–1212. doi: 10.1093/ajcn/nqz180

18. Wong VCH, Maguire JL, Omand JA, Dai DWH, Lebovic G, Parkin PC, O'Connor DL, Birken CS, Birken CS, Maguire JL, et al. A positive association between dietary intake of higher cow's milk-fat percentage and non−high-density lipoprotein cholesterol in young children. *J Pediatr* (2019) 211:105–111. doi: 10.1016/j.jpeds.2019.03.047

19. Kummer K, Jensen PN, Kratz M, Lemaitre RN, Howard B V, Cole SA, Fretts AM. Full-fat dairy food intake is associated with a lower risk of incident diabetes among American Indians with low total dairy food intake. *J Nutr* (2019) 149:1238–1244. doi: 10.1093/jn/nxz058

20. Laursen A, Sluijs I, Boer J, Verschuren W, van der Schouw Y, Jakobsen M. Substitutions between dairy products and risk of stroke: Results from the European Investigation into Cancer and Nutrition-Netherlands (EPIC-NL) cohort. *Br J Nutr* (2019) 121:1398–1404. doi: 10.1017/s0007114519000564

21. Johansson I, Esberg A, Nilsson LM, Jansson JH, Wennberg P, Winkvist A. Dairy product intake and cardiometabolic diseases in northern Sweden: A 33-year prospective cohort study.

Nutrients (2019) 11:284. doi: 10.3390/nu11020284

22. Koskinen TT, Virtanen HEK, Voutilainen S, Tuomainen TP, Mursu J, Virtanen JK. Intake of fermented and non-fermented dairy products and risk of incident CHD: The Kuopio Ischaemic Heart Disease Risk Factor Study. *Br J Nutr* (2018) 120:1288–1297. doi: 10.1017/s0007114518002830

23. Johansson I, Nilsson LM, Esberg A, Jansson JH, Winkvist A. Dairy intake revisited – associations between dairy intake and lifestyle related cardio-metabolic risk factors in a high milk consuming population. *Nutr J* (2018) 17:110. doi: 10.1186/s12937-018-0418-y

24. Dehghan M, Mente A, Rangarajan S, Sheridan P, Mohan V, Iqbal R, Gupta R, Lear S, Wentzel-Viljoen E, Avezum A, et al. Association of dairy intake with cardiovascular disease and mortality in 21 countries from five continents (PURE): a prospective cohort study. *Lancet* (2018) 392:2288–2297. doi: 10.1016/s0140-6736(18)31812-9

25. Sun Y, Magnussen CG, Dwyer T, Oddy WH, Venn AJ, Smith KJ. Cross-sectional associations between dietary fat-related behaviors and continuous metabolic syndrome score among young Australian adults. *Nutrients* (2018) 10:972. doi: 10.3390/nu10080972

26. Brouwer-Brolsma E, Sluik D, Singh-Povel C, Feskens E. Dairy product consumption is associated with pre-diabetes and newly diagnosed type 2 diabetes in the Lifelines Cohort Study. *Br J Nutr* (2018) 119:442–455. doi: 10.1017/S0007114517003762

27. Laursen ASD, Dahm CC, Johnsen SP, Tjønneland A, Overvad K, Jakobsen MU. Substitutions of dairy product intake and risk of stroke: A Danish cohort study. *Eur J Epidemiol* (2018) 33:201–212. doi: 10.1007/s10654-017-0271-x

28. Um CY, Judd SE, Flanders WD, Fedirko V, Bostick RM. Associations of calcium and dairy products with all-cause and cause-specific mortality in the REasons for Geographic and Racial Differences in Stroke (REGARDS) prospective cohort study. *Nutr Cancer* (2017) 69:1185–1195. doi: 10.1080/01635581.2017.1367946

29. Hruby A, Ma J, Rogers G, Meigs J, Jacques P. Associations of dairy intake with incident prediabetes or diabetes in middle-aged adults vary by both dairy type and glycemic status. *J Nutr* (2017) 147:1764–1775. doi: 10.3945/jn.117.253401

30. Guasch-Ferré M, Becerra-Tomás N, Ruiz-Canela M, Corella D, Schröder H, Estruch R, Ros E, Arós F, Gómez-Gracia E, Fiol M, et al. Total and subtypes of dietary fat intake and risk of type 2 diabetes mellitus in the Prevención con Dieta Mediterránea (PREDIMED) study. *Am J Clin Nutr* (2017) 105:723–735. doi: 10.3945/ajcn.116.142034

31. Karatzi K, Aissopou E, Tsirimiagou C, Fatmeli E, Sfikakis P, Protogerou A. Association of consumption of dairy products and meat with retinal vessel calibers in subjects at increased cardiovascular risk. *Nutr Metab Cardiovasc Dis* (2016) 26:752–757. doi: 10.1016/j.numecd.2016.03.006

32. Brouwer-Brolsma EM, van Woudenbergh GJ, Oude Elferink SJWH, Singh-Povel CM, Hofman A,

Dehghan A, Franco OH, Feskens EJM. Intake of different types of dairy and its prospective association with risk of type 2 diabetes: The Rotterdam Study. *Nutr Metab Cardiovasc Dis* (2016) 26:987–995. doi: 10.1016/j.numecd.2016.08.003

33. Santiago, Sayón-Orea C, Babio N, Ruiz-Canela M, Martí A, Corella D, Estruch R, Fitó M, Aros F, Ros E, et al. Yogurt consumption and abdominal obesity reversion in the PREDIMED study. *Nutr Metab Cardiovasc Dis* (2016) 26:468–475. doi: 10.1016/j.numecd.2015.11.012

34. Díaz-López A, Bulló M, Martínez-González M, Corella D, Estruch R, Fitó M, Gómez-Gracia E, Fiol M, García de la Corte F, Ros E, et al. Dairy product consumption and risk of type 2 diabetes in an elderly Spanish Mediterranean population at high cardiovascular risk. *Eur J Nutr* (2016) 55:349–360. doi: 10.1007/s00394-015-0855-8

35. Drehmer M, Pereira M, Schmidt M, Alvim S, Lotufo P, Luft V, Duncan B. Total and full-fat, but not low-fat, dairy product intakes are inversely associated with metabolic syndrome in adults. *J Nutr* (2016) 146:81–89. doi: 10.3945/jn.115.220699

36. Hosseinpour-Niazi S, Mirmiran P, Hosseini-Esfahani F, Azizi F. Is the metabolic syndrome inversely associates with butter, non-hydrogenated- and hydrogenated-vegetable oils consumption: Tehran lipid and glucose study. *Diabetes Res Clin Pract* (2016) 112:20–29. doi: 10.1016/j.diabres.2015.11.008

37. Drehmer M, Pereira MA, Schmidt MI, Molina MDCB, Alvim S, Lotufo PA, Duncan BB. Associations of dairy intake with glycemia and insulinemia, independent of obesity, in Brazilian adults: The Brazilian Longitudinal Study of Adult Health (ELSA-Brasil). *Am J Clin Nutr* (2015) 101:775–782. doi: 10.3945/ajcn.114.102152

38. Ericson U, Hellstrand S, Brunkwall L, Schulz C, Sonestedt E, Wallström P, Gullberg B, Wirfält E, Orho-Melander M. Food sources of fat may clarify the inconsistent role of dietary fat intake for incidence of type 2 diabetes. *Am J Clin Nutr* (2015) 101:1065–1080. doi: 10.3945/ajcn.114.103010

39. Buijsse B, Boeing H, Drogan D, Schulze MB, Feskens EJ, Amiano P, Barricarte A, Clavel-Chapelon F, De Lauzon-Guillain B, Fagherazzi G, et al. Consumption of fatty foods and incident type 2 diabetes in populations from eight European countries. *Eur J Clin Nutr* (2015) 69:455–461. doi: 10.1038/ejcn.2014.249

40. Sayón-Orea C, Bes-Rastrollo M, Martí A, Pimenta AM, Martín-Calvo N, Martínez-González MA. Association between yogurt consumption and the risk of metabolic syndrome over 6 years in the SUN study. *BMC Public Health* (2015) 15:170. doi: 10.1186/s12889-015-1518-7

41. Crichton GE, Alkerwi A. Dairy food intake is positively associated with cardiovascular health: Findings from observation of cardiovascular risk factors in Luxembourg Study. *Nutr Res* (2014) 34:1036–1044. doi: 10.1016/j.nutres.2014.04.002

42. Crichton GE, Alkerwi A. Whole-fat dairy food intake is inversely associated with obesity prevalence: Findings from the observation of cardiovascular risk factors in Luxembourg Study. *Nutr Res* (2014) 34:936–943. doi: 10.1016/j.nutres.2014.07.014

43. Avalos EE, Barrett-Connor E, Kritz-Silverstein D, Wingard DL, Bergstrom JN, Al-Delaimy WK. Is dairy product consumption associated with the incidence of CHD? *Public Health Nutr* (2013) 16:2055–2063. doi: 10.1017/s1368980012004168

44. Patterson E, Larsson SC, Wolk A, Akesson A. Association between dairy food consumption and risk of myocardial infarction in women differs by type of dairy food. *J Nutr* (2013) 143:74–79. doi: 10.3945/jn.112.166330

45. Holmberg S, Thelin A. High dairy fat intake related to less central obesity: A male cohort study with 12 years' follow-up. *Scand J Prim Health Care* (2013) 31:89–94. doi: 10.3109/02813432.2012.757070

46. Scharf RJ, Demmer RT, DeBoer MD. Longitudinal evaluation of milk type consumed and weight status in preschoolers. *Arch Dis Child* (2013) 98:335. doi: 10.1136/archdischild-2012-302941
