# Supplementary material for: Full-fat dairy products and cardiometabolic health outcomes: Does the dairy-fat matrix matter?
Source: Front Nutr. 2024 Jul 29;11:1386257. doi: 10.3389/fnut.2024.1386257 (PMC11317386; doi:10.3389/fnut.2024.1386257)
Supplement: Supplementary file 3 [file Table_3.pdf]

Supplementary Material

Supplemental Table 3. Summary of randomized controlled trials (n = 13) evaluating the effects of dairy fat from milk, yogurt, cheese, and/or butter on cardiometabolic disease risk factors.

Reference	Population	Study design	Duration	Intervention
Dunne et al. (1)	Irish adults with body mass index ≥ 25 kg/m^2<br>n = 127<br>≥ 50 years	4-arm parallel	-Experimental: 6 weeks	Diets with ~40 g of dairy fat<br>-Full-fat cheese (34% fat; 120 g)<br>-Reduced-fat cheese and butter (~31% fat; 120 g and 21 g, respectively)
Rancourt-Bouchard et al. (2)	Canadian adults with high-normal daytime blood pressure<br>n = 53<br>18-75 years	3-arm crossover	-Experimental: 6 weeks<br>-Washout: 4-12 weeks	Isocaloric diets<br>-Low-fat milk (1%; 3 x 250 mL/d)<br>-High-fat cheese (31% fat; 1x 50 g /d)
Chen et al. (3)	Chinese women with waist circumference ≥ 90 cm and body mass index ≥ 28 kg/m^2<br>n = 92<br>36-66 years	2-arm parallel	-Experimental: 24 weeks	-Whole-fat yogurt (2.9% fat; 220 g/d)<br>-Whole milk (3.7% fat; 220 g/d)
Hansson et al. (4)	Norwegian adults<br>n = 47<br>18-70 years	4-arm crossover	-Experimental: 6 hours<br>-Washout: 2-5 weeks	Meals with 45 g of dairy fat, 84 g white toast, 20 g raspberry jam<br>-Butter (82% fat; 52 g)<br>-Cheese (38% fat; 113 g)
Feeney et al. (5)	Irish adults with body mass index ≥ 25 kg/m^2<br>n = 127	4-arm parallel	-Experimental: 6 weeks	Diets with ~40 g of dairy fat<br>-Full-fat cheese (34% fat; 120 g)

	≥ 50 years			-Reduced-fat cheese and butter (~31% fat; 120 g and 21 g, respectively)
Brassard et al. (6)	Canadian adults with abdominal obesity<br>n = 46<br>18-65 years	5-arm crossover	-Experimental: 4 weeks<br>-Washout: 4 weeks	Isocaloric diets<br>-Butter (32% fat in diet; 90 g/2500 kcal/d)<br>-Cheese (32% fat in diet; 48.9 g/2500 kcal/d)
Engel et al. (7)	Danish adults<br>n = 17<br>20-70 years	2-arm crossover	-Experimental: 3 weeks<br>-Washout: none	Isovolumetric treatments<br>-Whole milk (3.5% fat; 500 mL/d)<br>-Skim milk (0.1% fat; 500 mL/d)
Drouin-Chartier et al. (8)	Canadian adults<br>n = 43<br>18-65 years	3-arm crossover	-Experimental: 8 hours<br>-Washout: 14 days	Macronutrient matched isocaloric meals with bread, icing, fruit juice, and 33 g of dairy fat/1000 kcal<br>-Cheddar cheese (32% fat)<br>-Cream cheese (31% fat)<br>-Butter
Brassard et al. (9)	Canadian adults with abdominal obesity<br>n = 92<br>18-65 years	5-arm crossover	-Experimental: 4 weeks<br>-Washout: 4 weeks	Isocaloric diets<br>-Butter (32% fat in diet; 90 g/2500 kcal/d)<br>-Cheese (32% fat in diet; 48.9 g/2500 kcal/d)
Raziani et al. (10)	Danish adults at risk of metabolic syndrome<br>n = 139<br>18-70 years	3-arm parallel	-Experimental: 12 weeks	-High-fat cheese (25%/32% fat; 80 g/2000 kcal/d)<br>-Reduced fat cheese (13%/16% fat; 80 g/2000 kcal/d)
Villalpando et al. (11)	Mexican children<br>n = 462; interventions randomized by boarding school<br>6-16 years	3-arm parallel	-Experimental: 4 months	Isovolumetric treatments<br>-Whole milk (3% fat; 400mL/d)<br>-Skim milk (0.5% fat; 400mL/d)
Loria-Kohen et al. (12)	Spanish adults<br>n = 161<br>25-65 years	2-arm parallel	-Experimental: 12 months	Isovolumetric treatments<br>-Semi-skim milk (~1.9% fat; 500 mL/d)<br>-Skim milk (~0.3% fat; 500 mL/d)

Penedo et al. (13)	Brazilian adults<br>n = 29<br>20-40 years	1-arm	-Depletion: 8 weeks<br>-Repletion: 8 weeks	Depletion-repletion design<br>-No dairy fat consumption<br>-Habitual dairy fat consumption with an additional 20 g/d butter

References

1. Dunne S, McGillicuddy FC, Gibney ER, Feeney EL. Role of food matrix in modulating dairy fat induced changes in lipoprotein particle size distribution in a human intervention. *Am J Clin Nutr* (2023) 117:111–120. doi: 10.1016/j.ajcnut.2022.10.002

2. Rancourt-Bouchard M, Gigleux I, Guay V, Charest A, Saint-Gelais D, Vuillemard J, Lamarche B, Couture P. Effects of regular-fat and low-fat dairy consumption on daytime ambulatory blood pressure and other cardiometabolic risk factors: A randomized controlled feeding trial. *Am J Clin Nutr* (2020) 111:42–51. doi: 10.1093/ajcn/nqz251

3. Chen Y, Feng R, Yang X, Dai J, Huang M, Ji X, Li Y, Okekunle AP, Gao G, Onwuka JU, et al. Yogurt improves insulin resistance and liver fat in obese women with nonalcoholic fatty liver disease and metabolic syndrome: A randomized controlled trial. *Am J Clin Nutr* (2019) 109:1611–1619. doi: 10.1093/ajcn/nqy358

4. Hansson P, Holven KB, Oyri LKL, Brekke HK, Biong AS, Gjevestad GO, Raza GS, Herzig K-H, Thoresen M, Ulven SM. Meals with similar fat content from different dairy products induce different postprandial triglyceride responses in healthy adults: A randomized controlled cross-over trial. *J Nutr* (2018) 149:422–431. doi: 10.1093/jn/nxy291

5. Feeney E, Barron R, Dible V, Hamilton Z, Power Y, Tanner L, Flynn C, Bouchier P, Beresford T, Noronha N, et al. Dairy matrix effects: Response to consumption of dairy fat differs when eaten within the cheese matrix-A randomized controlled trial. *Am J Clin Nutr* (2018) 108:1–8. doi: 10.1093/ajcn/nqy146

6. Brassard D, Arsenaultm B, Boyer M, Bernic D, Tessier-Grenier M, Talbot D, Tremblay A, Levy E, Asztalos B, Jones P, et al. Saturated fats from butter but not from cheese increase HDL-mediated cholesterol efflux capacity from J774 macrophages in men and women with abdominal obesity. *J Nutr* (2018) 148:573–580. doi: 10.1093/jn/nxy014

7. Engel S, Elhauge M, Tholstrup T. Effect of whole milk compared with skimmed milk on fasting blood lipids in healthy adults: A 3-week randomized crossover study. *Eur J Clin Nutr* (2018) 72:249–254. doi: 10.1038/s41430-017-0042-5

8. Drouin-Chartier JP, Tremblay AJ, Maltais-Giguère J, Charest A, Guinot L, Rioux LE, Labrie S, Britten M, Lamarche B, Turgeon SL, et al. Differential impact of the cheese matrix on the postprandial lipid response: A randomized, crossover, controlled trial. *Am J Clin Nutr* (2017) 106:1358–1365. doi: 10.3945/ajcn.117.165027

9. Brassard D, Tessier-Grenier M, Allaire J, Rajendiran E, She Y, Ramprasath V, Gigleux I, Talbot D, Levy E, Tremblay A, et al. Comparison of the impact of SFAs from cheese and butter on cardiometabolic risk factors: A randomized controlled trial. *Am J Clin Nutr* (2017) 105:800–809. doi: 10.3945/ajcn.116.150300

10. Raziani F, Tholstrup T, Kristensen M, Svanegaard M, Ritz C, Astrup A, Raben A. High intake of

regular-fat cheese compared with reduced-fat cheese does not affect LDL cholesterol or risk markers of the metabolic syndrome: A randomized controlled trial. *Am J Clin Nutr* (2016) 104:973–981. doi: 10.3945/ajcn.116.134932

11. Villalpando S, Lara Zamudio Y, Shamah-Levy T, Mundo-Rosas V, Manzano AC, Lamadrid-Figueroa H. Substitution of whole cows' milk with defatted milk for 4 months reduced serum total cholesterol, HDL-cholesterol and total apoB in a sample of Mexican school-age children (6-16 years of age). *Br J Nutr* (2015) 114:788–795. doi: 10.1017/s0007114515002330

12. Loria-Kohen V, Espinosa-Salinas I, Ramirez de Molina A, Casas-Agustench P, Herranz J, Molina S, Fonollá J, Olivares M, Lara-Villoslada F, Reglero G, et al. A genetic variant of PPARA modulates cardiovascular risk biomarkers after milk consumption. *Nutrition* (2014) 30:1144–1150. doi: 10.1016/j.nut.2014.02.012

13. Penedo LA, Nunes JC, Gama MAÔS, Leite PEC, Quirico-Santos TF, Torres AG. Intake of butter naturally enriched with cis9,trans11 conjugated linoleic acid reduces systemic inflammatory mediators in healthy young adults. *J Nutr Biochem* (2013) 24:2144–2151. doi: 10.1016/j.jnutbio.2013.08.006
