# Supplementary material for: Full-fat dairy products and cardiometabolic health outcomes: Does the dairy-fat matrix matter?
Source: Front Nutr. 2024 Jul 29;11:1386257. doi: 10.3389/fnut.2024.1386257 (PMC11317386; doi:10.3389/fnut.2024.1386257)
Supplement: Supplementary file 4 [file Table_4.pdf]

frontiers

Supplementary Material

Supplemental Table 4. Summary of results from observational studies presented in tables 1-5.

	Obesity	T2D[a]	CVD[b]	Inflammation	MetS[c]	Total
Regular-fat milk only	12	21	20	2	3	58
↔[d]	4	14	13	1	2	34 (59%)
↓[e]	7	5	2	1	1	16 (28%)
↑[f]	1	2	5	-[g]	-	8 (14%)
Regular-fat milk as a substitute	-	-	1	-	-	1
↔	-	-	1	-	-	1 (100%)
↓	-	-	-	-	-	-
↑	-	-	-	-	-	-
Regular-fat yogurt only	5	7	6	-	3	21
↔	2	4	5	-	3	14 (67%)
↓	3	2	1	-	-	6 (29%)
↑	-	1	-	-	-	1 (5%)
Regular-fat yogurt as a substitute	-	2	6	-	-	8
↔	-	1	3	-	-	4 (50%)
↓	-	1	3	-	-	4 (50%)
↑	-	-	-	-	-	-
Regular-fat cheese only	4	10	16	2	2	34
↔	2	6	8	1	2	19 (56%)
↓	1	3	5	1	-	10 (29%)
↑	1	1	3	-	-	5 (15%)

Butter only	3	11	16	1	4	35
↔	2	7	9	1	2	21 (60%)
↓	1	1	2	-	1	5 (14%)
↑	-	3	5	-	1	9 (26%)

[a]T2D = type 2 diabetes. [b]CVD = cardiovascular diseases. [c]MetS = metabolic syndrome. [d]No disease risk indicated. [e]Decreased disease risk indicated. [f]Increased disease risk indicated. [g]Outcome measure(s) not evaluated.
