# Supplementary material for: Full-fat dairy products and cardiometabolic health outcomes: Does the dairy-fat matrix matter?
Source: Front Nutr. 2024 Jul 29;11:1386257. doi: 10.3389/fnut.2024.1386257 (PMC11317386; doi:10.3389/fnut.2024.1386257)
Supplement: Supplementary file 5 [file Table_5.pdf]

frontiers

Supplementary Material

Supplemental Table 5. Summary of results from randomized controlled trials presented in tables 6 and 7.

	Obesity	Hyperglycemia and insulin resistance	Inflammation	Hypertension	Dyslipidemia	Total
Regular-fat milk compared to lower-fat milk	2	2	1	1	6	12
↔[a]	2	2	1	1	3	9 (75%)
↓[b]	-[c]	-	-	-	2	2 (17%)
↑[d]	-	-	-	-	1	1 (8%)
Regular-fat yogurt compared to other dairy products	1	2	2	1	1	7
↔	-	1	1	1	1	4 (57%)
↓	1	1	1	-	-	3 (43%)
↑	-	-	-	-	-	-
Regular-fat cheese compared to lower-fat cheese	1	1	1	-	3	6
↔	1	1	1	-	2	5 (83%)
↓	-	-	-	-	-	-
↑	-	-	-	-	1	1 (17%)
Regular-fat cheese compared to other dairy products	3	6	4	3	14	30
↔	3	5	4	3	8	23 (77%)
↓	-	-	-	-	4	4 (13%)
↑	-	1	-	-	2	3 (10%)
Butter intake compared to lower butter intake	1	1	2	-	-	4

↔	1	1	1	-	-	3 (75%)
↓	-	-	1	-	-	1 (25%)
↑	-	-	-	-	-	-

[a]No disease risk indicated. [b]Decreased disease risk indicated. [c]Outcome measure(s) not evaluated. [d]Increased disease risk indicated.
