# Supplementary material for: Full-fat dairy products and cardiometabolic health outcomes: Does the dairy-fat matrix matter?
Source: Front Nutr. 2024 Jul 29;11:1386257. doi: 10.3389/fnut.2024.1386257 (PMC11317386; doi:10.3389/fnut.2024.1386257)
Supplement: Supplementary file 6 [file Table_6.pdf]

Supplementary Material

Supplemental Table 6. Summary of results from all study designs.

		No disease risk	Decreased disease risk	Increased disease risk	Total
**Regular-fat dairy products total**		**137 (63%)**	**28 (13%)**	**51 (24%)**	**216**
**Milk total**		**44 (62%)**	**9 (13%)**	**18 (25%)**	**71**
Observational studies	Regular-fat milk only	34	8	16	58
Observational studies	Regular-fat milk as a substitute	1	-[a]	-	1
RCTs[b]	Regular-fat milk compared to lower-fat milk	9	1	2	12
**Yogurt total**		**22 (61%)**	**1(3%)**	**13 (36%)**	**36**
Observational studies	Regular-fat yogurt only	14	1	6	21
Observational studies	Regular-fat yogurt as a substitute	4	-	4	8
RCTs	Regular-fat yogurt compared to other dairy products	4	-	3	7
**Cheese total**		**47 (67%)**	**9 (13%)**	**14 (20%)**	**70**
Observational studies	Regular-fat cheese only	19	5	10	34
RCTs	Regular-fat cheese compared to lower-fat cheese	5	1	-	6
RCTs	Regular-fat cheese compared to other dairy products	23	3	4	30
**Butter total**		**24 (62%)**	**9 (23%)**	**6 (15%)**	**39**
Observational studies	Butter only	21	9	5	35
RCTs	Butter intake compared to lower butter intake	3	-	1	4

[a]Outcome measure(s) not evaluated. [b]Randomized controlled trials.
